# Relaxin-2-secreting CAR-T cells exhibit enhanced efficacy in stromal-rich xenograft tumors

**DOI:** 10.3389/fimmu.2025.1506204

**Published:** 2025-07-01

**Authors:** Hisataka Ogawa, Tomoya Ekawa, Yu Mizote, Takashi Akazawa, Hideaki Tahara

**Affiliations:** ^1^ Nitto Joint Research Department for Nucleic Acid Medicine, Research Center, Osaka International Cancer Institute, Osaka, Japan; ^2^ Department of Cancer Drug Discovery and Development, Research Center, Osaka International Cancer Institute, Osaka, Japan; ^3^ Center for Clinical Research, Osaka International Cancer Institute, Osaka, Japan

**Keywords:** chimeric antigen receptor T cell, pancreatic cancer, solid tumor, tumor stroma, relaxin-2, fibrosis, matrix metalloproteinase

## Abstract

**Introduction:**

Chimeric antigen receptor (CAR)-T cell therapy has demonstrated notable efficacy in treating hematological malignancies. Although it has shown promise in the clinical management of solid tumors, poor outcomes in clinical trials highlight the challenges in developing therapies suitable for the distinct tumor microenvironment, which features a dense stroma composed of fibroblasts and extracellular matrix proteins, such as collagen, hyaluronan, proteoglycans, laminin, and elastin. These predominantly fibroblast-produced components create a barrier that can impede CAR-T cell infiltration into tumors, limiting their efficacy. CAR-T cells that migrate from tumor vessels into the stroma may become trapped before reaching tumor cells.

**Methods:**

We engineered CAR-T cells to secrete relaxin-2 (RLN2), an antifibrotic peptide hormone. Known for its role in pregnancy, RLN2 facilitates the softening and remodeling of collagen in the cervix and pelvic ligaments, and also promotes collagen degradation in the tumor microenvironment by upregulating matrix metalloproteinase levels by binding to the receptor LGR7/RXFP1.

**Results:**

*In vitro* studies revealed that cancer cells exposed to CAR-T cell-secreted RLN2 exhibited an increased expression and secretion of specific matrix metalloproteinases. In mouse xenograft models with abundant stromal content, RLN2-secreting CAR-T cells demonstrated significantly improved antitumor efficacy and infiltration into the tumor microenvironment compared to conventional CAR-T cells.

**Discussion:**

RLN2 may enhance the antitumor activity of CAR-T cells against solid tumors by promoting their infiltration into the tumor microenvironment.

## 1 Introduction

Clinical data for hematological malignancies and clinical trials in solid tumors indicate that CD19 chimeric antigen receptor (CAR)-T cell therapy shows limited efficacy against solid tumors (1). The resistance of solid tumors to CAR T-cell therapy has been extensively studied, and the immunosuppressive tumor microenvironment has been identified as a major contributor (2, 3).

Current clinical trials on pancreatic and biliary tract cancers have focused on preventing the exhaustion of CAR-T cells primarily induced by the immunosuppressive environment (4, 5). Recent strategies have targeted histological structural abnormalities in solid tumors using adhesion molecules as antigens (6, 7). This approach aims to reduce side effects on normal tissues while allowing larger doses of CAR-T cells maximize efficacy. However, dense, desmoplastic, and fibrotic tumor stroma poses a substantial challenge bycan collapseing tumor blood vessels and physically separat the cancer nests, thereby hindering the delivery of therapeutics to cancer cells (8). We hypothesized that altering the physical stromal barrier produced by the thick tumor stroma could reopen the collapsed tumor vessels and facilitate CAR-T cell access to the cancer nests, This approach is crucial for enabling CAR-T cells towhere they can exert their cytolytic activity. However, the key stromal components responsible for establishing this physical barrier remain largely unelucidated.

Numerous studies have investigated the roles of heparan sulfate and hyaluronic acid, among other stromal components, in the effectiveness of novel CAR-T cell therapies (9, 10). Some approaches have been developed to target cancer-associated fibroblasts (CAFs), a major constituents of the tumor stroma (11–13). These strategies aim to disrupt the stromal barrier and enhance the infiltration and efficacy of CAR-T cells within the tumor microenvironment.

Among the components of the tumor stroma, we focused on collagen fibers. The basement membrane, primarily composed of type IV collagen, plays a crucial role in maintaining the integrity of the tumor vasculature and regulating immune cell extravasation into the tumor. Collagen types I and III contribute to the structural integrity and stiffness of the tumor stroma. This dense collagen network provides mechanical support and creates a physical barrier restricting CAR-T cell migration toward cancer cells (8).

To target these collagens, we focused on relaxin-2 (RLN2), a physiological peptide hormone of the RLN family that was initially discovered to participate in pregnancy. RLN2 is mainly produced and released by the corpus luteum and placenta during pregnancy and plays a crucial role in uterine labor (14, 15). RLN2 stimulates the production and release of nitric oxide within the uterine tissues by binding to its cognate receptor, leucine-rich repeat-containing G-protein coupled receptor 7/RLN family peptide receptor 1 (LGR7/RXFP1), leading to uterine muscle relaxation and vasodilation. Additionally, RLN2 stimulates the production of matrix metalloproteinases (MMPs), including MMP-1, -2, -3, -7, -9, -13, and -14—involved in collagen remodeling (16–18).

Given that RLN2 induces the expression of multiple MMPs, including MMP-2, -7, and -9, which mainly degrade collagen type IV, and MMP-1, -3, -13, and -14, which mainly degrade collagen types I and III, we aimed to harness RLN2-expressing CAR-T cells oversecreting MMPs. Because intratumoral MMP activity is tightly regulated by a complex network involving multiple MMPs and their natural inhibitors (tissue inhibitors of metalloproteinases), the induction of a broad spectrum of MMPs may be more effective than a single MMP (19), potentially overcoming compensatory mechanisms within the tumor microenvironment, leading to more favorable therapeutic outcomes.

To evaluate the role of the physiological stromal barrier in solid tumors, we developed a third-generation transposon-based CAR-T cell therapy targeting CD44 variant 6 (CD44v6) and evaluated its antitumor effects in xenograft models with both scant and abundant stroma. Anticipating the antifibrotic function of RLN2, we engineered RLN2-oversecreting CAR-T cells and evaluated their antitumor effects in xenograft models with abundant stroma compared the those of conventional CAR-T cell therapy.

## 2 Materials and methods

### 2.1 Cell lines and human peripheral blood mononuclear cells

This study utilized human pancreatic cancer cell lines SU86.86, Panc-1, MIA PaCa-2, and AsPC-1 [American Type Culture Collection (ATCC), Manassas, VA, USA]; human bile duct cancer cell lines TFK-1, HuCCT-1, and NOZ (ATCC); human colon cancer cell lines HT29, HCT116, and SW620 (ATCC); human breast cancer cell line MCF-7 (ATCC); human lung cancer cell line H522 (ATCC); human gastric cancer cell line MKN45 (ATCC); human sarcoma cell lines MG63 and HT1080 (ATCC); human prostate cancer cell line LNCap (ATCC); mouse NIH/3T3 (Japanese Collection of Research Bioresources, Osaka, Japan); immortalized human pancreatic stellate cells (imhPSC; Dr. Kenoki Ohuchida of Kushu University) (20); and KT86/64 [K562 engineered to express CD86 and a high-affinity Fc receptor (CD64)] (21). Fresh PBMCs were isolated from healthy volunteer blood or blood donated at the Japanese Red Cross Blood Center (Osaka, Japan) using Ficoll density centrifugation. All cell lines, except NIH/3T3, MG63, HT1080, and Phoenix AMPHO (ATCC), were cultured in Roswell Park Memorial Institute (RPMI)-1640 medium (Sigma Aldrich, St. Louis, MO, USA) supplemented with 10% fetal bovine serum (FBS; Biowest, Nuaillé, France) at 37°C with 5% CO_2_. NIH/3T3, MG63, HT1080, and Phoenix AMPHO cells were cultured in high-glucose Dulbecco’s modified Eagle’s medium (DMEM), and imhPSCs were cultured in DMEM/F12 (Sigma-Aldrich) supplemented with 10% FBS at 37°C with 5% CO_2_. All cell lines tested negative for *Mycoplasma* contamination (MycoAlert PLUS^™^, Lonza, Rockland, ME, USA). The experimental protocols adhered to the institutional guidelines.

### 2.2 Plasmid construction

Our third-generation Sleeping Beauty CAR construct design for *in vitro* and *in vivo* experiments is presented in **Supplementary Figure 1A**. A CAR construct targeting CD44v6, which included the leader sequence and VH-VL chains connected by a linker protein, was synthesized using a CD44v6 monoclonal antibody (22, 23). This construct was fused in-frame to the human IgG1-CH2-CH3 spacer and endo-domains, incorporating the CD28, 4-1BB, and CD3zeta domains (**Supplementary Figure 1A**) (24). For the *in vivo* experiments, the partial CH2 portion was omitted to prevent non-specific activation through interactions between Fc and FcR-bearing myeloid cells (25, 26). The non-viral vector pSBbi-GP (gifted by Eric Kowarz; Addgene plasmid # 60511) (27), was used as the base plasmid. The codon-optimized CAR construct was amplified by polymerase chain reaction (PCR) using primers containing SfiI cloning sites. The pSBbi-GP vector was digested with SfiI, and the amplified codon-optimized CAR construct was inserted to generate the pSBbi CAR CD44v6 plasmid. In certain experiments, enhanced green fluorescent protein (eGFP) was deleted from this plasmid and replaced with either modified firefly luciferase (Luc2) or human RLN2. We employed a similar strategy to develop a CAR construct directed against Claudin-4, which is overexpressed in several epithelial cancers (28). The human RLN2 cDNA sequence was obtained from the UniProt database (UniProt ID: P04090). All genetic manipulations to obtain CAR constructs were performed by GenScript (Piscataway, NJ, USA; Tokyo; Japan). The transposon was electroporated into fresh PBMCs along with the transposase pCMV(CAT)T7-SB100 (gifted by Zsuzsanna Izsvak; Addgene plasmid # 34879) (29).

A CD44v6-expressing MSCV retroviral vector was constructed to target the full-length CD44v6 (30). It was inserted in frame into the CD44v6-IRES-DsRed-Express2-Puromycin-resistance retroviral vector and introduced into AsPC-1 cells (31). The AsPC-1-CD44v6 cells were sorted and selected using puromycin. A retroviral vector construct was created and confirmed by DNA sequencing using Vector Builder Inc. Panc-1 cells were transduced with Luc-GFP-expressing lentivirus (PLV-10172-50, Cellomics Technology) along with polybrene, sorted, and selected with puromycin to generate Panc-1-Luc cells.

### 2.3 Generation of CD44v6 CAR-T and control T cells

On day 1, fresh PBMCs were subjected to 2 h of plastic adhesion for monocyte depletion and 10^6^ cells were combined with 100 µL of Ingenio^®^ Electroporation Solution (Mirus Bio, Madison, WI, USA), 9.5 µg of the CAR plasmid, and 0.5 µg of pCMV(CAT)T7-SB100 plasmid. Electroporation was performed using the T-023 program on a Nucleofector II electroporator (Amaxa, Cologne, Germany). After 1–2 h, the electroporated PBMCs were stimulated with irradiated OKT3-loaded KT86/64 cells at a ratio of 2:1 (viable PBMCs: OKT3-loaded KT86/64 cells) in RPMI-1640 or CTS OpTmizer T-Cell Expansion SFM (Gibco, Grand Island, NY, USA) supplemented with 10 ng/mL recombinant interleukin 15 (IL-15) and 20 ng/mL recombinant IL-21 (Peprotech, Rocky Hill, NJ, USA) (32–35). Fresh IL-15 and IL-21 were replaced every other day with puromycin selection (2.0 µg/mL) starting on day 6. On day 12, the selected CAR-T cells were either used for further experiments or cryopreserved using CELLBANKER 1 (Zenogen Pharma, Fukushima, Japan) at −80°C (**Supplementary Figure 1B**). As a control, 10^6^ PBMCs electroporated without the CAR plasmid were stimulated with irradiated OKT3-loaded KT86/64 cells at a 2:1 ratio (referred to as Ctrl-T cells or Ctrl-T).

### 2.4 Flow cytometry

Flow cytometry was performed using monoclonal antibodies conjugated to FITC, PE, BV421, APC, and APC/Cy7 (eBioscience and BioLegend, San Diego, CA, USA) according to the manufacturers’ instructions. The expression of CD44v6 in various cancer cell lines was assessed using a mouse anti-human FITC-conjugated CD44v6 antibody (clone VFF-18, eBioscience). The expression of claudin-4 in SU86.86 and H522 was assessed using a rabbit anti-human claudin-4 antibody (cat no. 16195-1-AP, Proteintech). Memory/effector phenotypes of CAR-T cells were monitored using CD45RO, CD62L, and CD3 antibodies, whereas degranulation and activation were detected using CD107a, CD69, and CD8a antibodies and the Golgi blocker monensin (1,000x; BioLegend). The memory/effector phenotypes were defined as naïve (CD62L^+^ CD45RO^-^), central memory (CD62L^+^ CD45RO^+^), effector memory (CD62L^-^ CD45RO^+^), and effector subsets (CD62L^-^ CD45RO^-^). CAR expression was confirmed using a phycoerythrin (PE)-conjugated Fc gamma-specific antibody (Jackson ImmunoResearch, West Grove, PA, USA). We used antibodies against CD3 (clone OKT3), CD4 (clone SK3), CD8a (clone RPA-T8), CD62L (clone DREG-56), CD45RO (clone UCHL1), CD69 (clone FN50), and CD107a (clones H4A3 or H130) (eBioscience and BioLegend). Data were collected using an Attune flow cytometer (Applied Biosystems, Carlsbad, CA, USA) or SA3800 spectral analyzer (Sony, Tokyo, Japan) and were subsequently analyzed.

### 2.5 Differentiation assay

Various cancer cell lines (0.5 × 10^6^ cells) were seeded into a 6-well tissue culture plate for 16–18 h before being co-incubated with Ctrl- or CAR-T cells in RPMI-1640 supplemented with 10% FBS without cytokines at a 1:1 effector-to-target (E:T) ratio. The co-culture was maintained at 37°C with 5% CO_2_ for 3 d. Surviving T cells were analyzed for their differentiation status by flow cytometry using the SA3800 spectral analyzer (Sony).

### 2.6 Cytotoxicity assay

Various cancer cell lines (ranging from 0.5 × 10^4^ to 1 × 10^4^) were seeded into a 96-well tissue culture plate for 16–18 h before being co-incubated with Ctrl- or CAR-T cells in RPMI-1640 with 10% FBS, without cytokines, at various ratios, and maintained at 37°C with 5% CO_2_ for 3 d. Surviving cancer cells were assessed using a crystal violet assay (36). Relative absorbance was calculated by dividing the absorbance of each well by the average absorbance of the wells containing untreated cancer cells, measured using an Infinite 200 Pro (Tecan Japan, Kanagawa, Japan).

### 2.7 Immunofluorescence staining

Panc-1 and NIH/3T3 cells (2 × 10^4^) were cultured in Millicell EZ SLIDE 8-well chamber slides (Millipore) for 16–18 h before being co-cultured with CAR-T cells at a ratio of 4:1 for 3 h at 37°C with 5% CO_2_. The cells were fixed with 4% paraformaldehyde for 10 min at 25°C, washed three times with PBS prepared with distilled water, and incubated in blocking buffer (PBS containing 1% bovine serum albumin and 0.1% Triton X-100) for 30 min at 25°C. The cells were then incubated for 16–18 h at 4°C with mouse anti-human CD44v6 (1:500; eBioscience) and rat anti-human CD3 (1:500; Abcam, Cambridge, UK) antibodies. After three washes with PBS, the cells were incubated with goat anti-mouse IgG1 secondary antibody (Alexa Fluor 568) and goat anti-rat IgG (H+L) secondary antibody (Alexa Fluor 488; Invitrogen, Waltham, MA, USA) at a ratio of 1:1,000 for 60 min in the dark. After washing, the slides were mounted using ProLong Gold Antifade Mountant with 4′,6-diamidino-2-phenylindole (DAPI, Invitrogen), and images were captured using a BZ-X800 microscope (Keyence, Tokyo, Japan).

### 2.8 CAR-T cell activation and degranulation assay

Each cancer cell line (2 × 10^4^) was cultured with CAR-T cells at a 2:1 ratio in 96-well plates. The CD107a antibody and Golgi blocker monensin were added to each well. After 20 h, the cells were harvested, stained with CD69 and CD8a antibodies, and analyzed using the SA3800 spectral analyzer (Sony).

### 2.9 Enzyme-linked immunosorbent assay

ELISAs for RLN2, interferon γ (IFN-γ), and tumor necrosis factor α (TNF-α) were performed using a Human RLN2 Quantikine ELISA Kit (DRL200, R&D Systems, Minneapolis, MN, USA), Human IFN Gamma/IFNG/Interferon Gamma ELISA Kit PicoKine^®^ (EK0373, Boster Bio, Pleasanton, CA, USA), and LEGEND MAX™ Human TNF-α ELISA (430207, BioLegend), following the manufacturers’ instructions. CAR-T (effector) cells were co-cultured with target tumor cells at defined E:T ratios—either 1:1 or 4:1 CAR-T cells per tumor cell—for 48 h. After incubation, supernatants were collected following centrifugation to remove cell debris and analyzed according to the ELISA kit protocols.

### 2.10 Gelatin zymography

As previously described (37), MMP activity was assessed using a gelatin zymography kit (AK-47; Cosmo Bio, Tokyo, Japan) following the manufacturer’s instructions. After washing thrice with serum-free RPMI-1640, Su86.86 cells were incubated in either serum-free RPMI-1640 or 10% FBS-supplemented RPMI-1640; the supernatant was collected from 1 × 10^6^/mL CAR-T or RLN2-secreting CAR-T cells for 48 h. Cell-free supernatants were analyzed for the presence of gelatinases to measure MMP activity. Gelatinolytic bands were visualized using a ChemiDoc MP imaging system.

### 2.11 RNA extraction and quantitative reverse transcription PCR

Total RNA was isolated from the cell lysates using an RNeasy Plus Mini Kit (Qiagen, Hilden, Germany) according to the manufacturer’s protocol. After measuring RNA concentration and quality, 500 µg of RNA was reverse transcribed into cDNA using SuperScript^™^ VILO^™^ Master Mix (Thermo Fisher Scientific, Waltham, MA, USA) in a T100 Thermal Cycler (Bio-Rad Laboratories, Hercules, CA, USA), following the manufacturer’s instructions. qPCR was performed using SYBR Green qPCR Master Mix (Thermo Fisher Scientific) according to the manufacturer’s protocol and under the following conditions: 95°C for 10 min, 45 cycles at 95°C for 15 s, 64°C for 30 s, and 75°C for 30 s for detection, followed by 95°C for 15 s, 60°C for 60 s, 95°C for 15 s, and 60°C for 15 s for dissociation using a QuantStudio^™^ 3 Real-Time PCR System (Thermo Fisher Scientific). All gene levels were normalized to those of the internal control *GAPDH*, and fold change was calculated using the 2^−ΔΔCt^ method. The primer sequences are listed in **Supplementary Table 1**.

### 2.12 Western blotting

Denatured proteins (25 µg) were separated by electrophoresis on 10% Mini-PROTEAN TGX Precast Protein Gels and transferred onto polyvinylidene fluoride membranes (Mini PVDF Transfer Packs) using a Trans-Blot^®^ Turbo^™^ System (Bio-Rad). After blocking with 5% non-fat milk in Tris-buffered saline with Triton X-100 buffer for 1 h at 25°C, the membranes were incubated for 16–18 h at 4°C with a primary antibody (anti-human LGR7/RXFP1, 1:1,000; MAB8898, R&D Systems) or anti-human CD44v6 (1:1,000; eBioscience). After three washes, the membranes were incubated with goat anti-mouse (1:2,000) or anti-rabbit Ig-conjugated horseradish peroxidase secondary antibodies (1:5,000) at room temperature for 1 h. Protein quantities were determined using an ECL^™^ Prime Western Blotting System (GE Healthcare), and images were captured using the ChemiDoc MP imaging system. Anti-GAPDH antibody (ab8245, Abcam) was used as a loading control.

### 2.13 Generation of subcutaneous xenograft model

A suspension containing 5 × 10^6^ Panc-1, 3 × 10^6^ SU86.86, 2 × 10^6^ AsPC-1, 2 × 10^6^ AsPC-1-CD44v6, 5 × 10^6^ Capan-1, and 5 × 10^6^ BXPC-3 cells was prepared in 100 μL Matrigel GFR (Corning), diluted with serum-free RPMI-1640 (1:1), and subcutaneously injected into both lateral flanks of 6- to 10-week-old female NSG mice (Charles River, Wilmington, MA, USA) under isoflurane inhalation-induced anesthesia. Once the subcutaneous tumors reached 100–500 mm^3^, the tumor volume was calculated using the modified ellipsoid formula: V = ½(length × width × width). The mice were randomly divided into treatment groups. Each group received an intravenous injection of 10 × 10^6^ ctrl-T, CAR-T, or RLN2-secreting CAR-T cells via the tail vein. Tumor growth was monitored using calipers until the mice were euthanized.

### 2.14 *In vivo* bioluminescent imaging

An *in vivo* imaging system was used to detect the accumulation of Luc2-expressing CAR-T cells in xenograft tumors. Ten minutes after the intraperitoneal injection of 150 mg/kg d-luciferin (potassium salt) (Cayman Chemical), the mice were anesthetized with isoflurane, and bioluminescence was measured.

### 2.15 Tumor tissue preparation and staining

Xenograft tumor specimens were cryopreserved in an OCT embedding compound before being sectioned into 8 μm-thick slices using a cryostat and fixed with cold acetone for 10 min. The sections were washed three times with PBS-T (0.1% Triton X-100 in PBS), blocked with normal horse serum for 20 min at 25°C and incubated overnight at 4°C with rabbit anti-human CD3 antibody (1:100; ab11089, Abcam), mouse anti-human CD44v6 (1:200; BMS125, eBioscience), and rabbit anti-mouse CD31 antibody (1:200; AF3628, R&D). After washing thrice with PBS-T, the sections were incubated for 60 min at room temperature with the following secondary antibodies: goat anti-mouse IgG1 Alexa Fluor 568 (1:500; Thermo Fisher Scientific), goat anti-rabbit IgG (H+L) Alexa Fluor 488 (1:500; Thermo Fisher Scientific), and goat anti-rabbit IgG (H+L)-cross-adsorbed Alexa Fluor 405 (1:500; Thermo Fisher Scientific). The sections were rewashed with PBS-T and mounted using ProLong Gold Antifade Mountant containing DAPI. The stained sections were stored at 4°C in the dark until imaging was performed.

The xenograft tumor samples were also embedded in paraffin, sectioned at 4 μm thickness, and stained with hematoxylin and eosin (H&E). Immunohistochemical (IHC) staining was performed using a VECTASTAIN^®^ Elite^®^ ABC Universal PLUS Kit (PK-8200; Vector Labs, Newark, CA, USA) with heat-induced epitope retrieval (pH 6.0), following the manufacturer’s protocol. Primary antibodies against CD44v6 (1:200; BMS125, eBioscience) and type IV collagen (1:200; NB120-6586, Novus Bio, Centennial, CO, USA) were used. To detect collagen types I and III, a Picro-Sirius Red staining kit (PSR-1, ScyTek, West Logan, UT, USA) was used according to the manufacturer’s instructions. All images were captured using a VS200 Slide Scanner (Olympus, Tokyo, Japan).

### 2.16 Statistical analysis

Statistical analyses were performed using GraphPad Prism v9.5 (GraphPad Software, San Diego, CA, USA). Data are presented as means ± standard error of the mean. Student’s *t*-test was used to assess the statistical difference between groups, and two-way analysis of variance (ANOVA) was used to determine the statistical differences between multiple groups, with statistical significance set at *P* < 0.05.

## 3 Results

### 3.1 Generation of CD44v6-targeting CAR-T cells and modification to secrete RLN2

In a CAR construct expressing eGFP, we assessed transduction efficiency based on the flow cytometric analysis of eGFP. Due to the presence of a human IgG-derived Fc region in the CAR structure, its surface expression could also be detected using a PE-conjugated antibody specific to Fcγ receptors (**Supplementary Figure 1C**). Although the initial electroporation efficiency was approximately 10–15% a day after electroporation, approximately 95% of the expanded T cells were CAR-T cells, as detected by a PE-conjugated Fc gamma-specific antibody after puromycin selection on day 11 (**Supplementary Figure 1D**). Using our protocol, CAR-T cells were generated from 40 mL of peripheral blood collected from each of three donors. Based on the CD3^+^ cell yield and eGFP^+^ cell purity, a sufficient number of CAR-T cells with high purity—suitable for *in vivo* experiments—were obtained within ~2 weeks from the start of the manufacturing process (**Supplementary Figure 1E**).

We generated CD44v6-targeting CAR-T cells to assess the role of the physical stromal barrier in determining the *in vivo* efficacy of CAR-T cells in solid tumors. We screened for the expression of CD44v6 in various cell lines, including pancreatic, bile duct, colon, gastric, and breast cancer, and sarcoma cell lines, along with the mouse cell line NIH/3T3, using flow cytometry and western blotting. We identified SU86.86, Panc-1, MIA PaCa-2, TFK-1, HuCCT-1, NOZ, MKN45, HCT116, HT29, MG63, and HT1080 as CD44v6-positive cancer cell lines, whereas AsPC-1, SW620, MCF7, and NIH/3T3 cells were either CD44v6-weak or negative (**Supplementary Figures 2A, B**).

**Figure 1:**
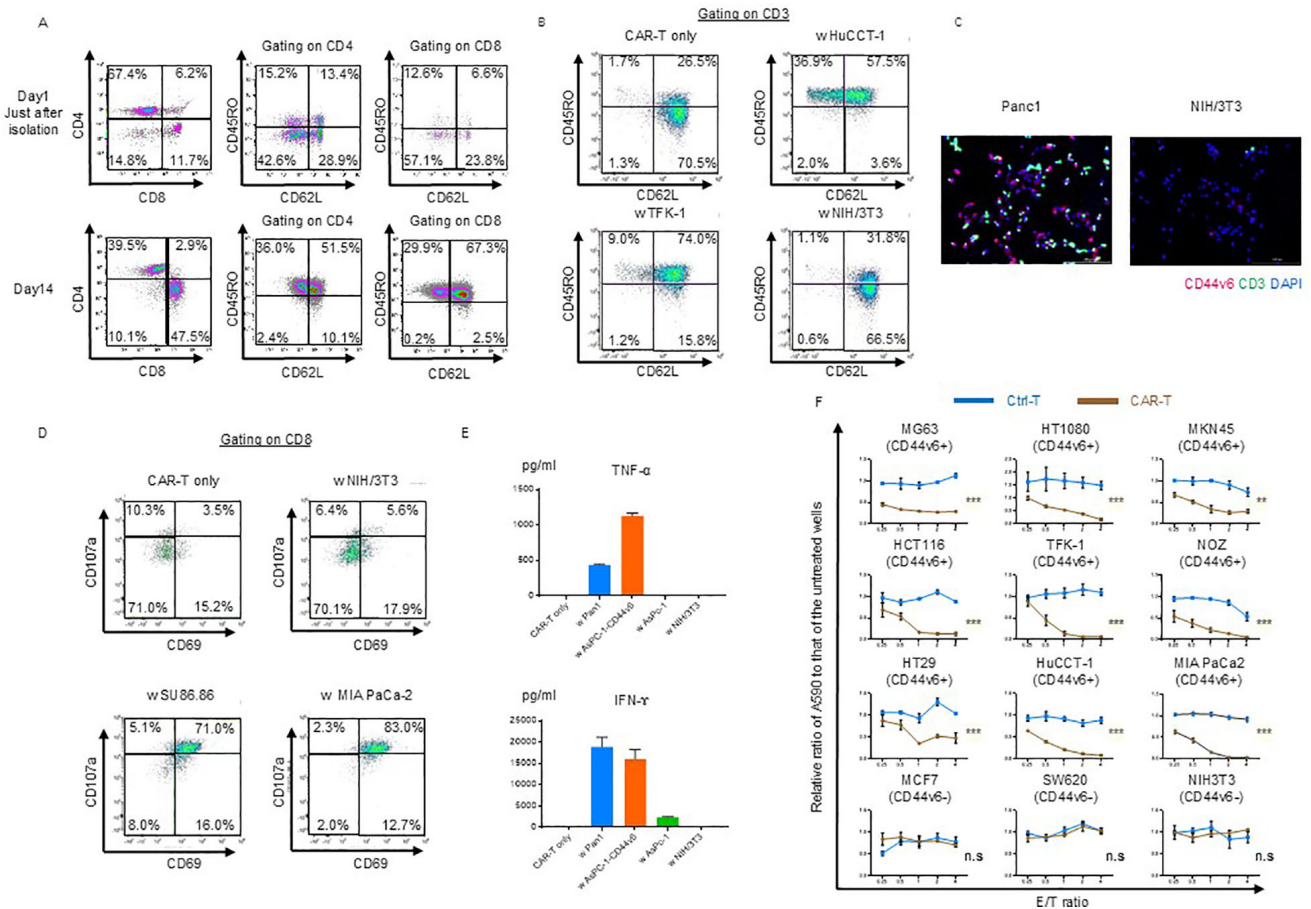
Phenotypic and functional characterization of CD44v6 chimeric antigen receptor (CAR)-T cells. **(A)** Memory/effector status of CAR-T cells before and after puromycin selection. Flow cytometry was performed using antibodies against the Fc gamma region, which binds to the hinge domain of the CAR expressed on both CD4^+^ and CD8^+^ T cells. Representative scatter plots are shown for donor 1-derived CAR-T cells. **(B)** Memory/effector subset analysis of CAR-T cells following co-culture with or without the indicated cell lines, followed by incubation in cytokine-depleted medium for 3 d Cells were stained with CD62L and CD45RO and gated on CD45^+^CD3^+^ CAR-T cells for flow cytometric analysis. Representative data from donor 1-derived CAR-T cells are shown. **(C)** Immunofluorescence analysis of antigen-dependent binding of CAR-T cells. Donor 1-derived CAR-T cells were co-cultured for 3 h with Panc-1 (CD44v6-positive) or NIH/3T3 (CD44v6-negative) cells, then stained with anti-CD3 antibody. Fluorescent labeling: CD3 (green), CD44v6 (red), and nuclei (DAPI, blue). **(D)** Antigen-dependent activation and degranulation of CAR-T cells. Cells were co-cultured for 24 h with or without target cell lines in cytokine-depleted medium in the presence of CD107a antibody and the Golgi transport inhibitor Monensin. Activation and degranulation were assessed using CD69 and CD8a staining. Representative flow cytometry plots are shown. **(E)** Cytokine secretion by CAR-T cells following co-culture with the indicated cell lines. Levels of TNF-α and IFN-γ were quantified by ELISA after 48 h at an E:T ratio of 1:1. Data are presented as mean ± standard deviation (SD) from three independent experiments using CAR-T cells derived from donors 1, 2, and 3. **(F)** Cytolytic activity of control T and donor 1-derived CAR-T cells against various cancer cell lines. Cells were co-cultured for 3 d and their viability was assessed using crystal violet staining. Absorbance values were normalized to untreated wells to calculate relative cancer cell survival. Data are presented as mean ± SD of three independent experiments performed in triplicate. ns, not significant; **P < 0.01; ***P < 0.001 (two-way ANOVA).

The generated CAR-T cell subsets were evaluated on day 14. Stimulation with OKT3-loaded KT86/64 cells at a 2:1 ratio, followed by subsequent culture with human IL-15 and IL-21 for 14 d, resulted in differentiation of naïve T cells into central and effector memory populations, with a minimal effector population (CAR-T only; **Figure 1A**). After incubation with NIH/3T3 cells (CD44v6-negative), most CAR-T cells remained in the naïve and central memory subsets (NIH/3T3, **Supplementary Figure 1B**). Although CAR-T cells showed further differentiation when co-cultured with CD44v6-positive cells (HuCCT-1 and TFK-1 cells), most remained in the central memory compartment (**Figure 1B**).

Using immunofluorescence staining, we confirmed the specific binding of CAR-T cells. After 3 h of co-culturing with Panc-1 and NIH/3T3 cells on chamber slides, the cells were fixed and stained with anti-CD44v6 and anti-CD3 antibodies to detect binding. The merged images demonstrated the antigen-specific binding of CAR-T cells (**Figure 1C**).

Cytotoxic T cell-mediated cytolysis involves degranulation and the release of cytotoxic cytokines such as IFN-γ and TNF-α. First, we assessed the extent of CAR-T cell activation and degranulation based on the expression of CD69 and CD107a. CD69 was partially upregulated in CAR-T cells on day 11 of expansion, whereas CD107a was barely detectable (**Figure 1D**). After incubation with NIH/3T3 cells (CD44v6 negative), degranulation was not induced via activation (NIH/3T3, **Figure 1D**). Only the co-culture with SU86.86 and MIA PaCa-2 (CD44v6-positive) induced activation and degranulation (**Figure 1D**).

We confirmed that TNF-α and IFN-γ were secreted during co-culture with Panc-1 and AsPC-1-CD44v6 cells (CD44v6-positive), whereas they were not detected during co-culture with AsPC-1 and NIH/3T3 cells (CD44v6-negative) (**Figure 1E**).

Finally, we evaluated the cytolytic ability of CD44v6-targeting CAR-T cells in coculture experiments and observed that it was exerted in an antigen- and dose-dependent manner (**Figure 1F**).

**Figure 2:**
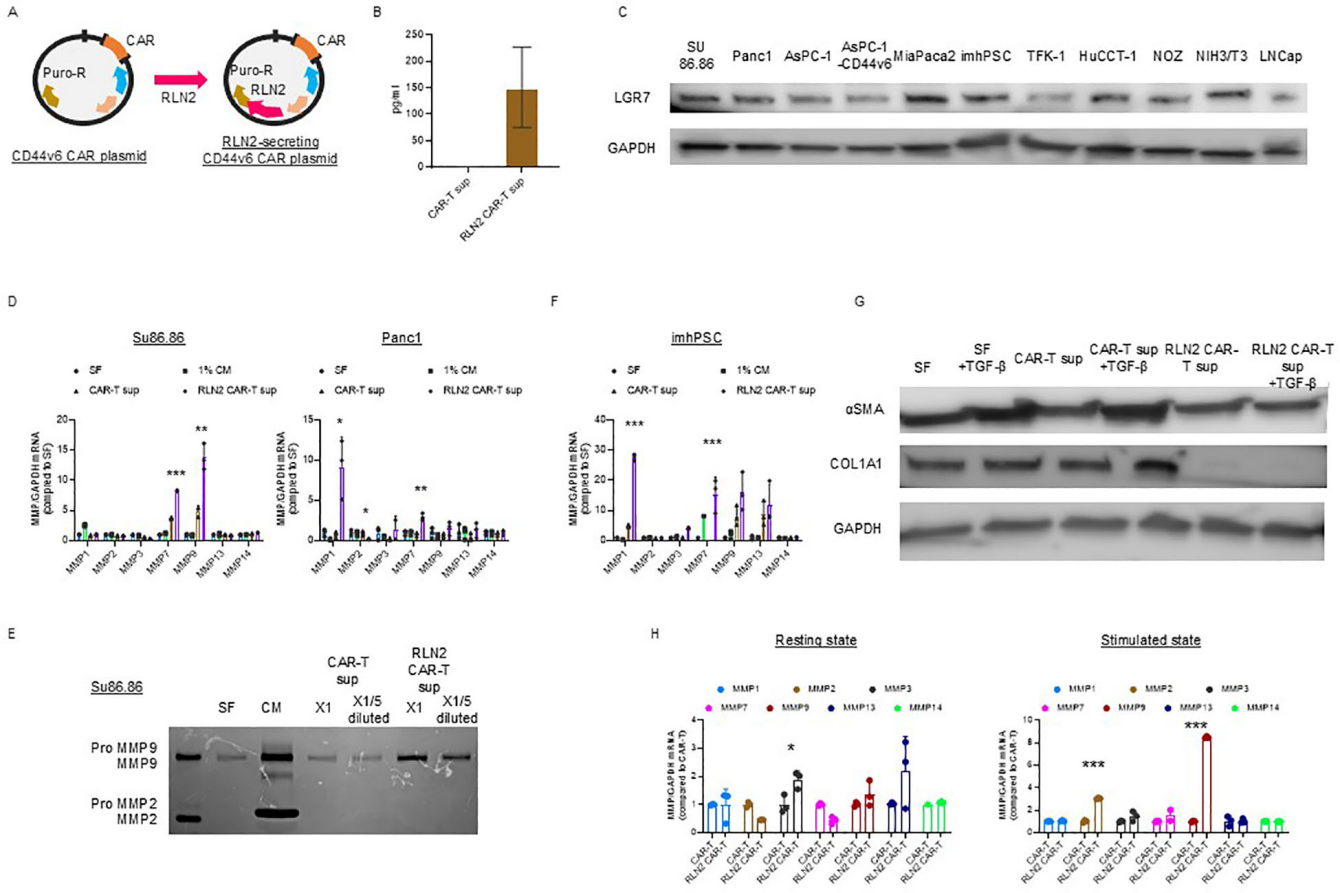
Development of CD44v6 CAR-T cells and engineering for RLN2 secretion. **(A)** Schematic illustration of plasmid constructs used for CAR-T cell generation, including CD44v6 CAR and co-expression of either Luc2 or human RLN2 via transposon-based vectors. **(B)** RLN2 protein levels secreted by conventional and RLN2-secreting CAR-T cells quantified using ELISA. Data are presented as mean ± SD from three independent experiments performed in triplicate using CAR-T cells derived from donors 1, 2, and 3. **(C)** Western blot analysis of the RLN2 receptor LGR7/RXFP1 in multiple cancer cell lines. GAPDH was used as a loading control. **(D)** Expression of MMPs in cancer cell lines cultured for 48 h in serum-free medium (SF), 1% fetal bovine serum (FBS)-containing medium (1% CM), supernatant from CAR-T cells (CAR-T sup), or supernatant from RLN2-secreting CAR-T cells (RLN2 CAR-T sup), collected after 24 h in SF. RT-qPCR was conducted in three independent runs using CAR-T cells from donors 1, 2, and 3, in triplicate (mean ± SD). *P < 0.05; **P < 0.01; ***P < 0.001; ns, not significant (one-way ANOVA followed by Bonferroni’s *post hoc* test and unpaired *t*-test for comparisons between CAR-T and RLN2-secreting CAR-T groups). **(E)** Gelatin zymography of conditioned media from SU86.86 cells cultured in SF, complete medium with 10% FBS (CM), or undiluted (×1) or 5-fold diluted (×1/5) supernatants from CAR-T or RLN2 CAR-T cells. **(F)** Western blot analysis of LGR7/RXFP1 expression in conventional and RLN2-secreting CAR-T cells. MMP expression in imhPSCs treated with the indicated supernatants (evaluated as described in **D**). **(G)** Western blot analysis of α-SMA and collagen type I alpha 1 (COL1A1) in imhPSCs cultured for 48 h in SF, SF + TGF-β (10 ng/mL), CAR-T sup ± TGF-β, or RLN2 CAR-T sup ± TGF-β. GAPDH was used as a loading control. Supernatants were derived from donor 1-generated CAR-T cells. **(H)** MMP expression in CAR-T cells. Conventional and RLN2-secreting CAR-T cells were cultured in RPMI-1640 with 10% FBS under resting conditions or with stimulation (anti-CD3/CD28 antibodies + IL-15 and IL-21) for 3 d RT-qPCR was performed using cells from donors 1, 2, and 3 (mean ± SD; triplicate experiments). *P < 0.05; ***P < 0.001; ns, not significant (comparisons between CAR-T and RLN2 CAR-T cells analyzed using unpaired *t*-test).

We hypothesized that the stromal barrier in tumors could be overcome by degrading collagen fibers via forced expression of human RLN2 in CAR-T cells (**Figure 2A**). We examined RLN2 secretion by the engineered CAR-T cells expressing human RLN2 (**Figure 2B**). Using ELISA, we confirmed that conventional CAR-T cells did not secrete RLN2, whereas RLN2-secreting CAR-T cells did (**Figure 2B**).

RLN binds to its G protein-coupled receptor, RLN family peptide receptor 1 (LGR7/RXFP1), to activate intracellular signaling and exert its effects (16). Therefore, we confirmed the expression of LGR7/RXFP1 in cancer cell lines using immunoblotting, including the human pancreatic cancer cell lines (SU86.86, Panc-1, AsPC-1, AsPC-1-CD44v6, and MIA PaCa-2 cells), bile duct cancer cell lines (TFK-1, HuCCT-1, and NOZ), imhPSCs, and LNCap (**Figure 2C**) (36).

We investigated changes in the expression of MMP-1, -2, -3, -7, -9, -13, and -14, known to be induced by RLN2 (17, 18, 37, 38), using RT-qPCR. After 16–18 h of culture in serum-free media, cell-free supernatants were collected from conventional and RLN2-secreting CAR-T cells. SU86.86 and Panc-1 cells were cultured in the supernatants for 2 d, and MMP expression was verified. Cancer cells cultured in serum-free medium alone were used as controls, and all experimental cells were cultured in a medium containing 1% FBS. Supernatants from RLN2-secreting CAR-T cells significantly induced MMP-7 and MMP-9 expression in SU86.86 cells and MMP-1 and MMP-7 in Panc-1 cells compared to those in conventional CAR-T cells (**Figure 2D**). Increased MMP-9 expression in SU86.86 cells was confirmed by gelatin zymography using cell culture supernatants. RLN2 from RLN2-secreting CAR-T cells increased active MMP-9 expression in SU86.86 cells, whereas conventional CAR-T cells did not induce MMP-9 expression (**Figure 2E**). We examined the effect of RLN2 on pancreatic stellate cells—considered major contributors to the desmoplastic reaction in human pancreatic cancer. Exposure of imhPSCs to RLN2 led to an upregulation of MMP-1 and MMP-7 mRNA expression (**Figure 2F**). Several studies have demonstrated that exposure of PSCs to transforming growth factor-β (TGF-β) induces their activation, as evidenced by increased α-smooth muscle actin (α-SMA) expression and extracellular matrix component production, particularly type I collagen. For instance, Apte et al. reported that α-SMA-positive PSCs co-localize with collagen type I-deposited areas in fibrotic pancreatic tissues, both in human chronic pancreatitis and experimental rat models, suggesting a central role for PSCs in pancreatic fibrosis (37). Furthermore, TGF-β1 stimulation has been shown to markedly increase the expression of α-SMA and collagen I in cultured PSCs, supporting its role as a key profibrotic cytokine in the pancreatic microenvironment (38). We next investigated whether RLN2 could counteract TGF-β–induced activation of imhPSCs, specifically α-SMA expression and collagen production. Stimulation with TGF-β led to a marked upregulation of α-SMA expression and collagen synthesis. Remarkably, however, exposure to RLN2 significantly suppressed both α-SMA expression and collagen production, even in the presence of TGF-β stimulation (**Figure 2G**).

T cells predominantly express MMP-2 and MMP-9, which play crucial roles in tissue infiltration and migration, respectively (39). We investigated the autocrine effects of RLN2 on the induction of MMP expression in CAR-T cells. To evaluate the differences in MMP expression based on T cell activation status, CAR-T and RLN2-secreting CAR-T cells were stimulated with 10 µg/mL soluble anti-CD3 antibody and 10 µg/mL anti-CD28 antibody for 3 d in the presence of IL-15 and IL-21. After activation, the cells were harvested, and the levels of MMPs were assessed using qPCR and compared with those in unstimulated resting cells to determine the differential expression of MMPs depending on the autocrine effects of RLN2. In the resting state, no significant increase in MMP expression induced by autocrine RLN2 signaling was observed in CAR-T cells, except for MMP-3 (left-resting state, **Figure 2H**). However, following stimulation, MMP-2 and MMP-9 expression in RLN2-secreting CAR-T cells increased significantly (right-stimulated state, **Figure 2H**).

We evaluated the cytolytic ability, differentiation status, and cytokine secretion capabilities of the RLN2-secreting CAR-T cells. In co-culture experiments with SU86.86, Panc-1, and AsPC-1-CD44v6 cells, RLN2-secreting CAR-T cells showed a cytotoxic ability similar to that of conventional CAR-T cells (**Supplementary Figure 3A**), and maintained an almost identical differentiation status after co-culture with SU86.86, Panc-1 (CD44v6-positive), and NIH/3T3 cells (CD44v6-negative) (**Supplementary Figure 3B**).

IFN-γ secretion levels were similar, whereas TNF-α secretion was significantly higher in RLN-secreting CAR-T cells than in conventional CAR-T cells. Notably, RLN2 secretion levels were approximately 2–3 times higher when RLN2-secreting CAR-T cells were stimulated with a co-culture of SU86.86 cells (CD44v6-positive) compared to no co-culture or co-culture with NIH/3T3 cells (CD44v6-negative) (**Supplementary Figure 3C**).

To account for donor variability, we evaluated the cytolytic activity and differentiation status of CAR-T and RLN2-secreting CAR-T cells generated from different donors. We observed no significant difference in cytolytic activity between conventional and RLN2-secreting CAR-T cells against SU86.86, Panc-1, and AsPC-1-CD44v6 cells, regardless of donor (**Supplementary Figure 4A**). Similarly, no marked differences were observed in the differentiation status of CAR-T cells across donor-derived products (**Supplementary Figure 4B**).

These results suggest that RLN2 from RLN2-secreting CAR-T cells promotes MMP expression via paracrine and autocrine signaling. This may enable RLN2-secreting CAR-T cells to penetrate the stromal barrier and more efficiently reach SU86.86 cells at the tumor site.

### 3.2 Histological characteristics of pancreatic cell line-derived subcutaneous xenograft tumors

We selected pancreatic cancer cell lines with abundant cancer-associated stroma, similar to the histological characteristics of clinical pancreatic adenocarcinomas, to assess the contributions of the stroma in solid tumors toward impairing the delivery of CAR-T cells to cancer cells.

Pancreatic adenocarcinoma is typically characterized by abundant stromal tissue due to desmoplastic changes, in which cancerous cells are surrounded by dense stroma. Histological analysis of pancreatic cancer samples resected at our facility revealed an abundant stromal component enveloping atypical glandular structures (data not shown). This stroma contains notable amounts of collagen fibers, particularly types I, III, and IV (40, 41).

We examined the histological characteristics of subcutaneous tumors derived from CD44v6-positive pancreatic cancer cell lines SU86.86, Panc-1, and AsPC-1-CD44v6.

**Figure 3:**
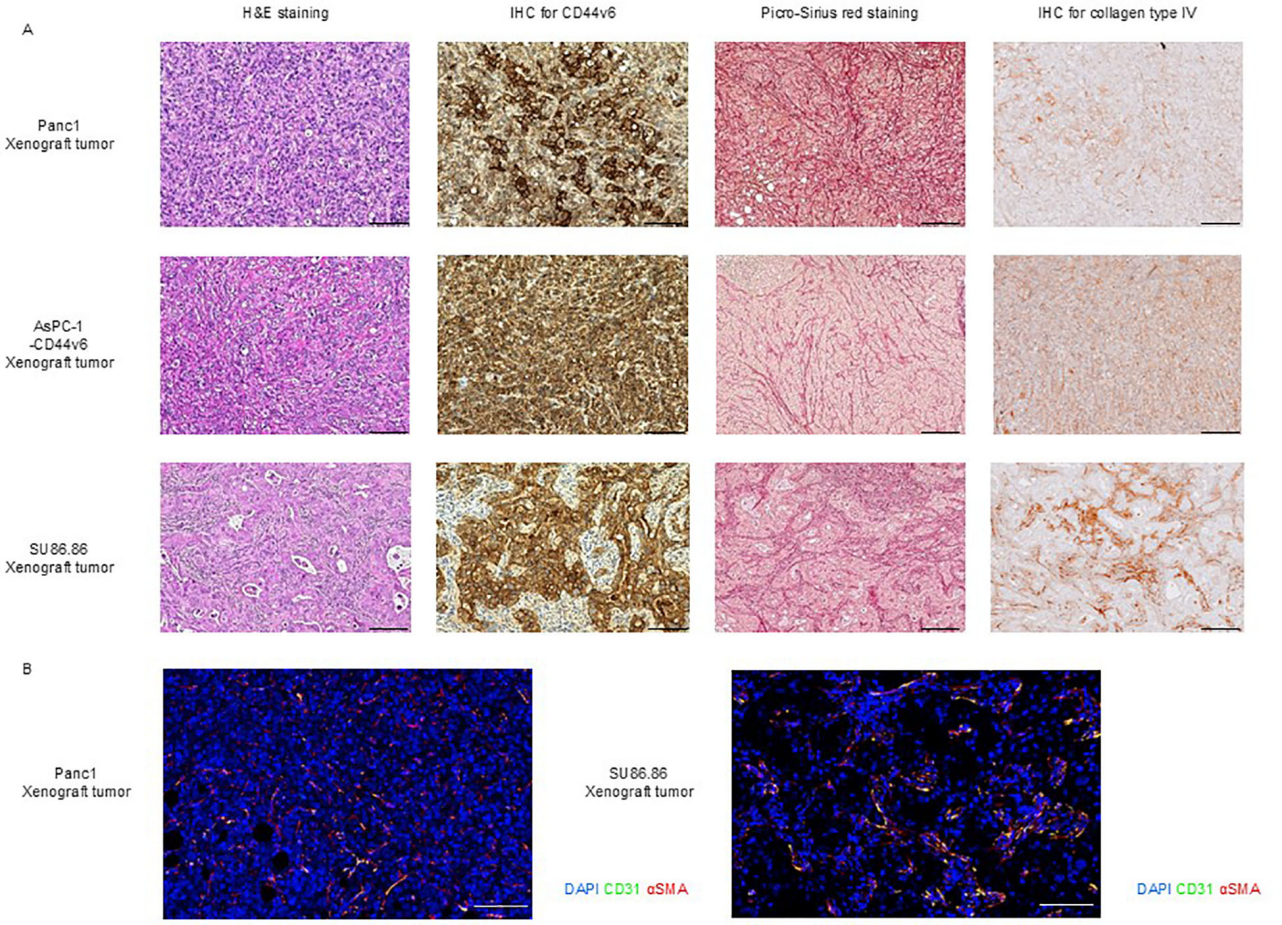
Histological characterization of subcutaneous xenograft tumor models. **(A)** Histological analysis of Panc-1, AsPC-1-CD44v6, and SU86.86 xenograft tumors. Tumor sections were analyzed for CD44v6 expression and stromal architecture. Formalin-fixed paraffin-embedded samples were subjected to H&E staining for tissue morphology, Picro-Sirius Red staining for collagen types I and III, and immunohistochemistry for CD44v6 and collagen type IV analysis. Scale bar = 200 µm. **(B)** Immunofluorescence staining of vascular and stromal markers in Panc-1 and SU86.86 subcutaneous xenograft tumors. Endothelial cells were labeled with anti-CD31 (green), myofibroblasts and pericytes with anti-α-SMA (red), and nuclei with DAPI (blue). Scale bar = 100 µm.

H&E staining of subcutaneous tumor from Panc-1 and AsPC-1-CD44v6 xenograft models revealed sarcoma-like features without the formation of distinct cancerous clusters. The stromal components were characterized by very fine collagen type I and type III fibers (as seen from Sirius Red staining) as well as collagen type IV fibers [IHC; upper, Panc-1; middle. AsPC-1-CD44v6; **Figure 3A**). In contrast, SU86.86 subcutaneous xenograft tumors exhibited a histological structure in which abundant stroma surrounded the cancerous clusters. Picro-Sirius Red staining showed that the stroma contained collagen types I and III, and IHC for collagen type IV revealed deposits around the clusters rich in these fibers (bottom in **Figure 3A**).

We confirmed that subcutaneous tumors from Panc-1, AsPC-1-CD44v6, and SU86.86 xenograft models were positive for CD44v6 (**Figure 3**), whereas subcutaneous tumors from the AsPC-1 xenograft model were negative for CD44v6 (**Supplementary Figure 5A**).

As reported by Smith et al. (42), subcutaneous tumor models derived from cancer cell lines can be broadly classified into two categories based on their vascular architecture: tumor and stroma vessel types. The latter more closely reflects the stromal-rich microenvironment observed in human pancreatic cancer specimens. However, only a limited number of pancreatic cancer cell lines are capable of forming stroma vessel-type tumors. As the efficacy of certain therapies—particularly those influenced by stromal barriers—may depend on tumor architecture, such treatments must be evaluated using appropriate tumor models.

To this end, we investigated the vascular classification of subcutaneous Panc-1 and SU86.86 xenograft tumors through immunohistochemical staining for CD31 (a marker of endothelial cells) and α-SMA (a marker of stromal myofibroblasts/pericytes). Based on the distribution of blood vessels and stromal components, Panc-1 tumors were categorized as tumor vessel type, whereas SU86.86 tumors exhibited features consistent with the intermediate type, as proposed in the original classification (**Figure 3B**) (42).

### 3.3 Conventional CAR-T cells are effective against xenograft tumors with scant but not abundant stroma

**Figure 4:**
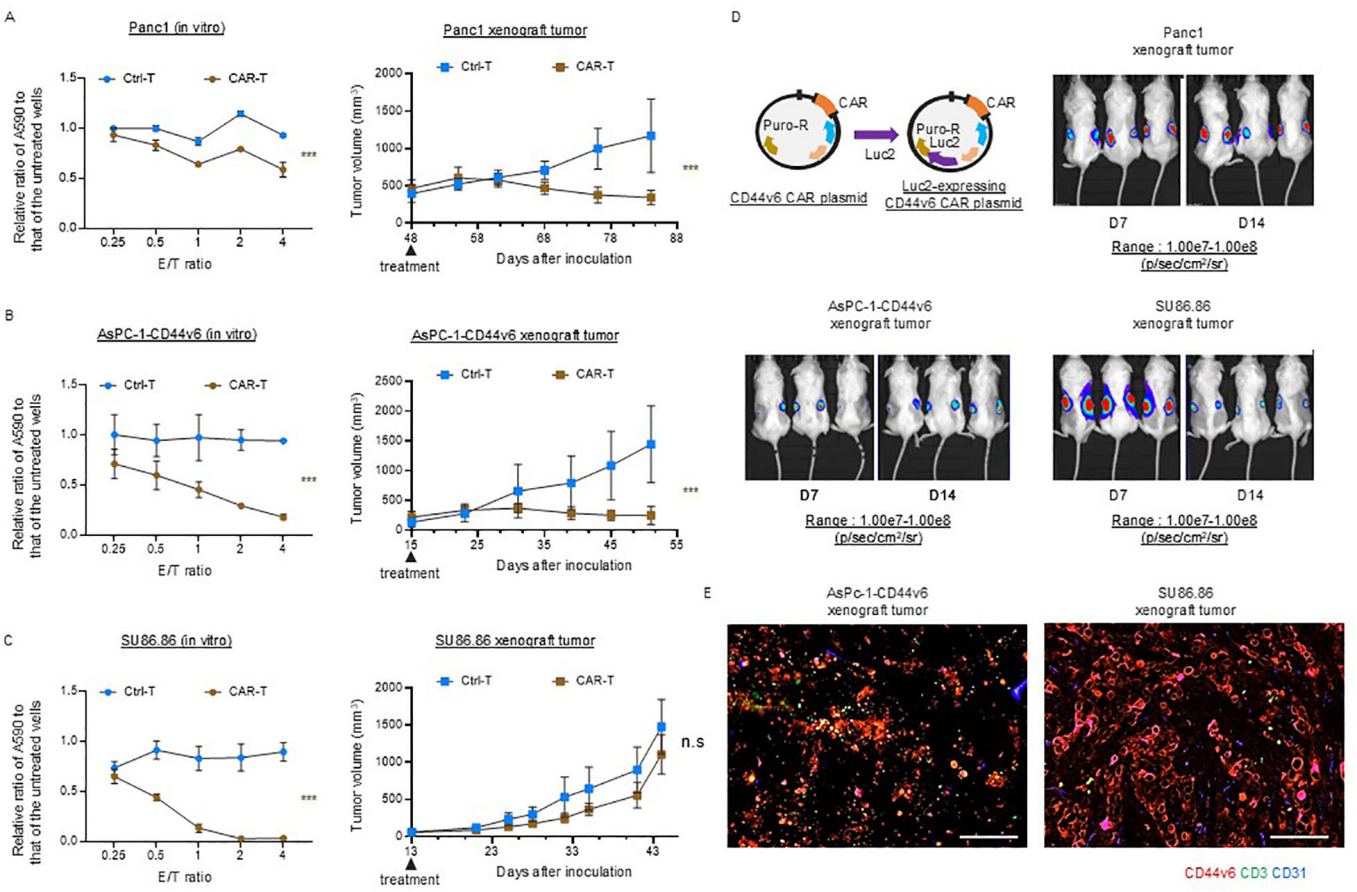
Efficacy of conventional CD44v6 CAR-T cell therapy in xenograft models with either scant or abundant stroma. **(A–C)** *In vitro* cytotoxicity (left) and *in vivo* antitumor activity (right) of donor 1-derived CD44v6 CAR-T cells against Panc-1 **(A)**, AsPC-1-CD44v6 **(B)**, and SU86.86 **(C)** tumor models. For *in vitro* assays, CAR-T cells were co-cultured with the indicated cancer cell lines for 3 d, and surviving cancer cells were quantified using a crystal violet assay. Absorbance at 590 nm (A_590_) was measured and normalized to untreated controls. Data are presented as mean ± SD from three independent experiments conducted in triplicate. ns, not significant; ***P < 0.001 (two-way ANOVA). For *in vivo* studies, tumor-bearing mice received a single intravenous injection of either Ctrl-T (*n* = 6) or CAR-T cells (*n* = 6). Data are presented as mean ± SD. ***P < 0.001; ns, not significant (two-way ANOVA). **(D)** *In vivo* bioluminescence imaging of Luc2-expressing CAR-T cell accumulation in subcutaneous xenograft tumors. A total of 1 × 10^7^ Luc2-expressing CAR-T cells were injected intravenously via the tail vein. Bioluminescence was measured using the IVIS imaging system on days 7 and 14 post-injection. **(E)** Immunofluorescence analysis of CAR-T cell infiltration in AsPC-1-CD44v6 and SU86.86 xenograft tumors 7 d after systemic injection of Luc2-expressing CAR-T cells. Tumor sections were stained for CD3 (green), CD44v6 (red), and CD31 (blue). Scale bar = 100 µm.

We investigated the efficacy of conventional CAR T-cell therapy in subcutaneous xenograft tumor models with scant and abundant stroma. First, we confirmed that the three CD44v6-positive cancer cell lines were similarly affected by the cytolytic activity *in vitro*, whereas no cytolytic activity was observed against CD44v6-negative AsPC-1 cells [Panc-1 (**Figure 4A**, left), AsPC-1-CD44v6 (**Figure 4B**, left), SU86.86 (**Figure 4C**, left)]. We then performed *in vivo* experiments. We administered 10^6^ viable Ctrl-T and CAR-T cells per mouse. AsPC-1-CD44v6, SU86.86, and AsPC-1 cells formed subcutaneous xenograft tumors of ~100 mm ~2 weeks after subcutaneous transplantation. In contrast, Panc-1 cells exhibit delayed tumor growth after engraftment. In preliminary experiments, CAR-T cells showed a potent antitumor effect when administered to Panc-1 subcutaneous tumors, approximately100 mm in size, resulting in tumor disappearance (data not shown). Therefore, we tested the antitumor effects of CAR-T cells in a larger Panc-1 subcutaneous xenograft tumor model (~500 mm). Once the tumors reached an appropriate size, the tumor-bearing NSG mice were randomly divided into two groups—the (1) Ctrl-T and (2) CAR T-cell groups—with a single intravenous injection administered via the tail vein. In both Panc-1 and AsPC-1-CD44v6 subcutaneous tumors, tumor growth ceased within 2 weeks of treatment and remained halted for ~1 month [Panc-1 (**Figure 4A**, right), AsPC-1-CD44v6 (**Figure 4B**, left)]. In contrast, AsPC-1 cells do not express CD44v6 and did not exert cytolytic activity *in vitro* (**Supplementary Figure 5B**). This lack of antigen expression was directly reflected *in vivo*, as CAR-T cells exhibited no antitumor effect against subcutaneous AsPC-1 tumors (**Supplementary Figure 5C**).

Notably, in SU86.86 subcutaneous tumors, conventional CAR-T cell therapy generated from the blood of donor 1 failed to exert a significant antitumor effect and did not halt tumor progression (**Figure 4C** left).

We investigated the differential sensitivity of AsPC-1-CD44v6 and SU86.86 subcutaneous tumors to CAR-T cell therapy based on the homing behavior of CAR-T cells. We monitored *in vivo* Luc2-expressing CAR-T cell distribution using an IVIS imaging system immediately after infusion and at 1 and 2 weeks post-injection (**Figure 4D**). Using the same scale range (1.00e7–1.00e8 p/s/cm²/sr) applied to Panc-1, AsPC-1-CD44v6, and SU86.86 models, we observed no detectable BLI signal in any organ, including the spleen, at 1 week. However, by 2 weeks, a signal was detected in the AsPC-1 tumor, indicating delayed CAR-T cell accumulation (**Supplementary Figure 5D**, left).

At a lower scale range (4.00e5–2.50e7 p/s/cm²/sr), CAR-T cell signals were clearly observed in the tumor and lungs at all time points (day 0, week 1, and week 2), suggesting that the cells persisted at low levels, below the detection threshold of the initial scale (**Supplementary Figure 5D**, right). The delayed CAR-T cell accumulation in AsPC-1 tumors may reflect chemokine-mediated recruitment by tumor-derived factors.

CAR-T cell accumulation remained stable between 1 and 2 weeks in Panc-1 and AsPC-1-CD44v6 tumors, while it declined in SU86.86 tumors, potentially due to stromal entrapment or immunosuppression within the tumor microenvironment.

We sampled AsPC-1-CD44v6 and Su86.86 xenograft tumors to examine the distribution of CAR-T cells. Immunofluorescence staining revealed that, in AsPC-1-CD44v6 xenograft tumors, the administered CAR-T cells were delivered to the tumor. Conversely, in SU86.86 xenograft tumors, CAR-T cells remained within the cancer stroma and were not efficiently delivered (**Figure 4E**). As hypothesized, these results suggest that the poor antitumor efficacy of CAR-T cell therapy in solid tumors may be due to stromal barriers impeding their inefficient delivery to the tumor.

### 3.4 RLN2-secreting CAR-T cells are effective in xenograft tumors with abundant stroma

**Figure 5:**
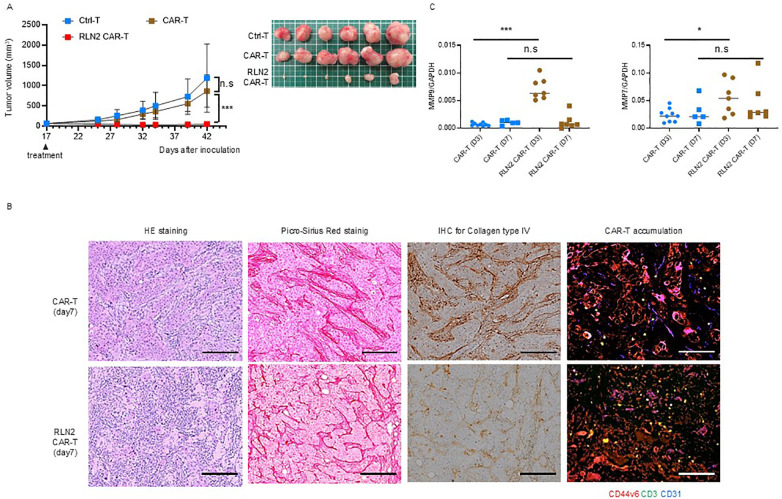
Antitumor effects of RLN2-secreting CD44v6 CAR-T cell therapy in stromal-rich SU86.86 xenograft tumors. **(A)** Tumor growth in SU86.86 subcutaneous xenografts after single intravenous injection of Ctrl-T (1 × 10^7^ cells, *n* = 6), conventional CD44v6 CAR-T (1 × 10^7^ cells, *n* = 6), or RLN2-secreting CD44v6 CAR-T cells (1 × 10^7^ cells, *n* = 6). Tumor size was measured over time, and representative images of resected tumors were captured following euthanasia. Data are presented as mean ± SD. ns, not significant; ***P < 0.001 (two-way ANOVA with Tukey’s multiple comparisons test). **(B)** Histological analysis of SU86.86 xenograft tumors collected 7 d after treatment with conventional or RLN2-secreting CAR-T cells. Formalin-fixed paraffin-embedded sections were subjected to H&E staining, Picro-Sirius Red staining (collagen types I and III), and immunohistochemistry. Immunofluorescence analysis of tumor-infiltrating CAR-T cells was performed on frozen sections. Tumor sections were stained for CD44v6 (red), CD3 (green), and CD31 (blue). Scale bar = 100 µm. **(C)** Quantitative analysis of MMP expression in SU86.86 xenograft tumors treated with conventional or RLN2-secreting CAR-T cells and harvested at either 3 or 7 d after single treatment with 1 × 10^7^ CAR-T cells. MMP-7 and MMP-9 mRNA levels were assessed using RT-qPCR. CAR-T group (*n* = 9, day 3; *n* = 5, day 7), RLN2 CAR-T group (*n* = 7, day 3; *n* = 7, day 7). Data from two independent experiments are shown as mean ± SD. ns, not significant; *P < 0.05; ***P < 0.001; ns, not significant (unpaired *t*-test).

qPCR analysis revealed the upregulation of MMP-9 and MMP-7 expression in SU86.86 cells *in vitro* in response to the supernatant from RLN2-secreting CAR-T cells. Gelatin zymography revealed the enhanced expression of active MMP-9. Considering that conventional CAR-T cells did not exhibit significant antitumor effects, we investigated the antitumor efficacy of RLN2-secreting CAR-T cells against SU86.86 subcutaneous xenograft tumors. When the tumors reached approximately 100 mm³, the mice were randomized into groups according to treatment: Ctrl-T cell infusion (Group 1), conventional CAR-T cell infusion (Group 2), and RLN2-secreting CAR-T cell infusion (Group 3). Tumor growth was periodically measured for approximately 1 month, and the tumor tissues were photographed following euthanasia. RLN2-secreting CAR-T cells from the blood of donor 1 significantly inhibited tumor growth compared to both conventional CAR-T and Ctrl-T cells (**Figure 5A**). Xenograft tumors were collected 7 d after treatment and histologically examined. In the conventional CAR-T cell group, cancer clusters remained intact, whereas they were disrupted in the RLN2-secreting CAR T-cell group, leading to a reduction in the number of cancer cells (**Figure 5B**). No appreciable differences were present in the deposition of collagen type I or type III between the conventional and RLN2-secreting CAR-T cell-treated groups (**Figure 5B**). Most notably, the collagen type IV staining intensity was markedly reduced in the RLN2-secreting CAR-T cell group compared to that in the conventional CAR-T cell group (**Figure 5B**). Immunofluorescence staining for CD3, used to detect tumor-infiltrating CAR-T cells, demonstrated that RLN2-secreting CAR-T cells were present not only in the stroma but also around the cancer cells within the tumor clusters. Conversely, conventional CAR-T cells were restricted to the stroma (**Figure 5B**). We investigated the induction of MMP expression by RLN2, as inferred *in vitro*, in tumors harvested 3 and 7 d after the infusion of conventional and RLN2-secreting CAR-T cells using qPCR. On day 3 post-treatment, the RLN2-secreting CAR-T cell group showed significantly increased expression of MMP-9 and MMP-7 compared with the conventional CAR-T cell group (**Figure 5C**). However, by day 7 post-treatment, no significant differences were observed between the two groups (**Figure 5C**). We evaluated the expression of other MMPs (MMP-1, MMP-2, MMP-3, MMP-13, and MMP-14) in these xenograft tumors but found no significant differences between the groups (**Supplementary Figure 6**). These results suggest that RLN2 from RLN2-secreting CAR-T cells induces the increased expression of MMP-9 and MMP-7 in SU86.86 cells *in vivo*. By preferentially degrading collagen type IV, in addition to collagen type I and III fibers, RLN2 overcomes the stromal barrier, enabling RLN2-secreting CAR-T cells to reach cancer cell nests and potentially enhance their cytolytic activity and antitumor efficacy.

**Figure 6:**
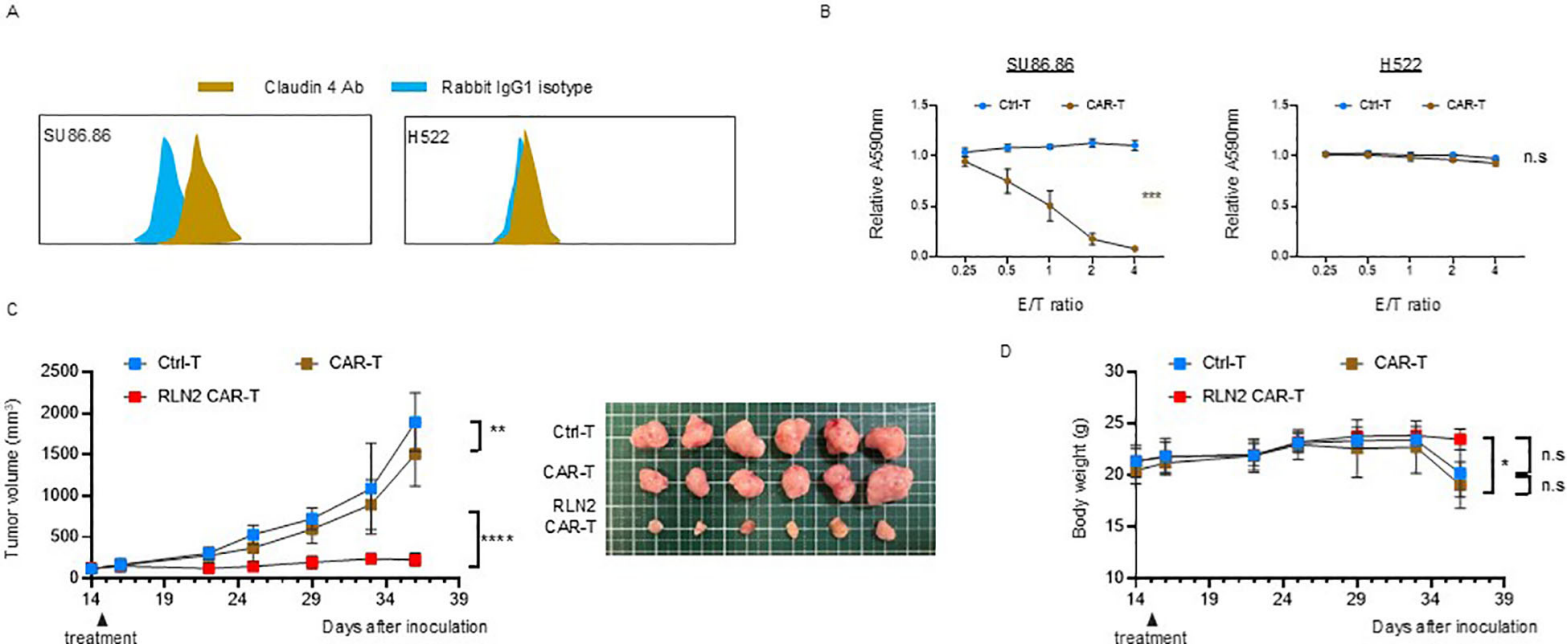
Antitumor efficacy of RLN2-secreting Claudin-4 CAR-T cell therapy in stromal-rich SU86.86 xenograft tumors. **(A)** Representative flow cytometry histograms of Claudin-4 expression in SU86.86 and H522 cells. Cells were stained with anti-Claudin-4 antibody (brown) or isotype control antibody (rabbit IgG1; blue). A rightward shift in fluorescence intensity indicates Claudin-4 expression in SU86.86 cells. **(B)** *In vitro* cytotoxicity of donor 3-derived Claudin-4 CAR-T and Ctrl-T cells against SU86.86 (Claudin-4^+^) and H522 (Claudin-4^-^) cells. Cells were co-cultured for 3 d, and cell viability was assessed using crystal violet staining. Absorbance values were normalized to untreated controls. Data are presented as mean ± SD from three independent experiments performed in triplicate. ns, not significant; ***P < 0.001 (two-way ANOVA). **(C)** *In vivo* tumor growth in SU86.86 xenograft models following single intravenous injection of Ctrl-T (1 × 10^7^ cells, *n* = 6), Claudin-4 CAR-T (1 × 10^7^ cells, *n* = 6), or RLN2-secreting Claudin-4 CAR-T cells (1 × 10^7^ cells, *n* = 6). Tumor volumes were measured longitudinally, and representative tumor images were obtained at the endpoint. Data are presented as mean ± SD. **P < 0.01; ****P < 0.0001 (two-way ANOVA with Tukey’s multiple comparisons test). **(D)** Body weight changes in each treatment group over the course of therapy. Data are shown as mean ± SD. ns, not significant; *P < 0.05 (two-way ANOVA with Tukey’s multiple comparisons test).

To further investigate whether the enhanced antitumor efficacy observed with RLN2-secreting CAR-T cells in the SU86.86 subcutaneous tumor model was dependent on the specific target antigen, we evaluated an alternative CAR construct targeting claudin-4, an adhesion molecule highly expressed on SU86.86 cells. We generated a claudin-4-specific CAR construct and validated its antigen specificity *in vitro* using SU86.86 cells (claudin-4^+^) and H522 lung cancer cells (claudin-4^-^) (**Figure 6A**). Cytotoxicity assays confirmed antigen-specific dose-dependent lysis of SU86.86 cells, with no significant cytolysis of claudin-4-negative H522 cells, confirming the specificity of the claudin-4 CAR construct (**Figure 6B**). Using this model, we evaluated the impact of RLN2 secretion on therapeutic efficacy in SU86.86 subcutaneous tumors. Similar to the results obtained with CD44v6-targeted CAR-T cells, RLN2-secreting claudin-4 CAR-T cells generated from the blood of donor 3 showed enhanced antitumor activity compared to their conventional CAR-T cells (**Figure 6C**).

Importantly, no evidence of toxicity or weight loss attributable to RLN2 secretion was observed in terms of mouse health status and body weight, indicating that RLN2 was safe for use *in vivo* (**Figure 6D**).

We sought to further evaluate the therapeutic potential of RLN2-secreting CAR-T cells in models that more closely recapitulate the stromal-rich tumor microenvironment encountered in human pancreatic cancer. Accordingly, we screened for models characterized with stroma vessel type tumors and identified two human pancreatic cancer cell lines, Capan-1 and BxPC-3, both of which exhibited high CD44v6 expression (**Supplementary Figure 7A**) and detectable LGR7 expression, the cognate receptor for RLN2 (**Supplementary Figure 7B**). Importantly, subcutaneous tumors derived from both cell lines were classified as stroma vessel type based on histological analysis (**Supplementary Figure 7C**).

Although both Capan-1 and BxPC-3 cells exhibited antigen-dependent and dose-dependent cytolytic activity *in vitro* (**Supplementary Figure 7D**, left), they were resistant to therapy using conventional CD44v6-targeted CAR-T cells from donor 1 *in vivo*, consistent with our hypothesis (**Supplementary Figure 7D**, right). *In vitro* cytotoxicity assays revealed no significant difference in the cytolytic activity of CAR-T cells against Capan-1 or BxPC-3 cells with or without RLN2 secretion (**Supplementary Figure 7E**, left). We evaluated the efficacy of RLN2-secreting CAR-T cells in these models however, observed that RLN2 secretion did not enhance antitumor activity in either tumor model (**Supplementary Figure 7E**, right).

## 4 Discussion

Pancreatic and biliary tract cancers are known for their poor prognoses and challenging treatment landscapes, requiring the urgent development of innovative therapeutic strategies. Despite promising results in *in vitro* studies and immunodeficient mouse models with cancer cell line-derived subcutaneous xenograft tumors, the same outcomes have not been consistently replicated in clinical trials of CAR-T cell therapy against solid tumors. During the time of this study, no CAR T-cell therapy had been clinically applied to these cancers. We hypothesized that this discrepancy is largely due to the substantial difference between cancer cell line-derived xenograft and actual patient tumor microenvironments. Specifically, several preclinical models exhibit a sarcoma-like histology with scant stroma, which may not accurately represent the dense stromal barrier present in clinical pancreatic and biliary tract cancers. Therefore, this study assessed the roles of stromal barriers in regulating CAR-T cell delivery to cancer cells and the potential of RLN2 expression and secretion by CAR-T cells for enhancing antitumor efficacy in pancreatic and biliary tract cancers.

To test our hypothesis, we developed transposon-based third-generation CAR-T cells targeting CD44v6. We employed a Sleeping Beauty-based vector because transposon-based gene delivery can stably integrate vectors into chromosomes, allowing for long-term expression with lower immunogenicity and a reduced risk of insertional mutagenesis (43). Although these transposon systems are less efficient than retroviral and lentiviral methods, we obtained a high-purity CAR-T cell population by employing puromycin-based selection and culture techniques (**Supplementary Figure 1D**). Our protocol, which utilizes OKT3-loaded antigen-presenting cells (21, 34) for stimulation instead of antibodies, produced CD44v6-targeted CAR-T cells exhibiting *in vitro* cytolytic activity (**Figure 1F**), cytokine production (**Figure 1E**), and memory cell fractions (**Figure 1A**), which were comparable to or exceeded those reported for other CAR-T cell therapies (23, 43).

Notably, the differentiation and exhaustion status of CAR-T cells are associated with their long-term persistence *in vivo* and antitumor efficacy (44). In our study, CAR-T cells developed using the Sleeping Beauty system demonstrated a predominance of memory stem cell fractions, even after co-culture with target antigens *in vitro* (**Figure 1B**), suggesting promising *in vivo* persistence for improved antitumor effects in mouse tumor models.

We used RLN2-secreting CAR-T cells to overcome stromal barriers. RLN2 is a pleiotropic peptide known for its NO-mediated vasodilatory effects on endothelial cells and exerts antifibrotic properties through the induction of MMPs in various stromal cells (45). Because of these functions, the development of RLN2 has advanced in non-oncology fields. Its vasodilatory effects have motivated its evaluation in phase 3 clinical trials as a recombinant protein for the treatment of acute heart failure (46, 47). Although the efficacy of RLN2 in acute heart failure has not been confirmed, various preclinical studies have confirmed its promising antifibrotic effects in fibrosis-related diseases, such as hypertrophic cardiomyopathy (48, 49), hepatic fibrosis (50), and renal fibrosis (48). Additionally, the recombinant protein showed minimal systemic side effects in clinical trials (51), suggesting its potential clinical application in various fibrotic conditions.

Systemic administration of human RLN2 in mice has been widely employed in fields outside of oncology, particularly in studies focusing on its antifibrotic effects. Notably, these studies have demonstrated that human RLN2 exhibits potent biological activity in mice through the activation of RXFP1 signaling, despite species differences in the RLN family (52). Preclinical studies consistently report that systemic administration of human RLN2 in mice does not cause organ toxicity or adverse effects, even at pharmacologically relevant doses.

Human RLN2 (serelaxin) has been administered systemically in humans during large-scale clinical trials for the treatment of acute heart failure, where its safety profile has been well established without significant toxicities (51). Both preclinical and clinical studies provide evidence on the pharmacological efficacy and favorable safety profile of human RLN2. RLN2 demonstrates promising characteristics for use in cancer therapy. Its antifibrotic properties can be harnessed to enhance oncolytic adenoviral therapies and improve cancer immunotherapy. Reports have indicated that introducing RLN2 into cancer cells may suppress fibrosis within tumors by modulating macrophage polarization, thereby enhancing cytotoxic T cell infiltration and immune checkpoint inhibitor efficacy (53).

The effects of RLN2 on MMP expression have previously been reported in breast cancer and glioblastoma cell lines (18, 54). While relaxin can upregulate MMPs in various tumor models, this does not necessarily translate into enhanced metastasis in all contexts. For example, Radestock et al. (55) demonstrated that long-term exposure of MDA-MB-231 breast cancer cells to relaxin resulted in reduced tumor growth and decreased cell motility and invasiveness, likely mediated through downregulation of S100A4 expression, despite the known MMP-inducing effects of relaxin. Hu et al. (56) demonstrated that relaxin gene delivery mitigated liver metastasis and synergized with checkpoint therapy by remodeling the tumor microenvironment to be less conducive to metastatic colonization. Therefore, while relaxin promotes MMP expression, its role in metastasis is not universally beneficial and may vary depending on tumor type, receptor expression, and tumor microenvironment. Although we did not conduct an evaluation of distant metastasis during the long-term follow-up period using the subcutaneous tumor model, our investigation revealed that the RLN2 receptor LGR7/RXFP1 was expressed in multiple pancreatic and biliary cancer cell lines (**Figure 2C**). In particular, RLN2 treatment led to the increased expression of MMP-7 and MMP-9 in Su86.86 cells, and MMP-1 and MMP-7 in Panc-1 cells (**Figure 2D**). In pancreatic stellate cells, the major sources of CAFs in pancreatic cancer, RLN2 induced MMP-1 and MMP-7 expression (**Figure 2F**).

RLN2 binds to its receptor LGR7/RXFP1 and activates various downstream signaling pathways, including cyclic adenosine monophosphate, phosphatidylinositol-4,5-bisphosphate 3-kinase, protein kinase B, extracellular signal-regulated kinase (ERK), NO, neuronal NO synthase (nNOS), cyclic guanosine monophosphate (cGMP), mitogen-activated protein kinases, and protein kinase C. These pathways exert a range of pleiotropic effects, such as vasodilatory, antifibrotic, angiogenic, anti-apoptotic, and anti-inflammatory effects, with different pathways involved depending on the cell type (17, 45).

The antifibrotic effects of RLN2 are mediated by crosstalk with other receptors. Particularly, sustained exposure to RLN2 inhibits TGF-β1 signaling-induced myofibroblast differentiation and collagen production by suppressing Smad2 phosphorylation via the phosphoERK-nNOS-NO-cGMP signaling pathway (16). Similarly, our study demonstrated that RLN2-secreting CAR-T cell supernatants suppressed TGF-β-induced α-SMA expression and collagen type I production in imhPSCs (**Figure 2G**).

We explored the autocrine effects of RLN2-secreting CAR-T cells on MMP expression. qPCR analyses revealed that RLN2-secreting CAR-T cells, particularly activated cells, induced MMP-2 and MMP-9 expression, though at lower levels than that in cancer cells, suggesting that the primary mechanism of stromal dissolution by RLN2-secreting CAR-T cells is paracrine, acting on cancer cells. Nevertheless, the observed increase in MMP-2 and MMP-9 expression in CAR-T cells may contribute to their migration from the tumor vasculature, highlighting their additional therapeutic role (39).

Even under non-stimulated conditions, RLN2 from RLN2-secreting CAR-T cells was sufficient to induce MMP expression in SU86.86 cells *in vitro*. Histological analysis showed that conventional CAR-T cells remained within the tumor stroma in the SU86.86 subcutaneous tumor model. Collectively, these findings suggest that RLN2 secreted locally by stromal CAR-T cells acts on SU86.86 tumor cells to enhance MMP expression, playing a pivotal role in the therapeutic effect. In contrast, the autocrine induction of MMPs within CAR-T cells may play a more supportive or secondary role in this context.

After the development of RLN2-secreting CAR-T cells and validation of their MMP-inducing capacity in various cancer cell lines and CAR-T cells *in vitro*, along with their basic phenotypes, we established and evaluated a stromal barrier model of subcutaneous tumors derived from various pancreatic cancer cell lines. As expected, histological examination showed that subcutaneous tumors derived from Panc-1 and AsPC-1-CD44v6 exhibited sarcoma-like histology with scant stromal components (**Figure 3**). However, SU86.86 subcutaneous xenograft tumors presented a unique histology characterized by cancer cell clusters surrounded by a thick abnormal stroma (**Figure 3**). This model provides a useful tool for assessing the impact of stromal barriers on CAR-T cell delivery.

Models that closely resemble the human tumor microenvironment are essential for better elucidating the histological features of pancreatic cancer. Patient-derived xenograft models, which involve the implantation of human tumor tissues into immunocompromised mice, and organoid-derived xenograft models, which use engineered tissue constructs, are notable for closely replicating the complex tissue architecture and stromal components found in clinical samples (57–59). For pancreatic cancer, co-injection models incorporating pancreatic stellate cells, either in the subcutaneous or orthotopic settings, have demonstrated a more accurate depiction of the fibrotic stroma than models using cancer cells alone (60–63). Orthotopic pancreatic cancer models with forced expression of TGF-β1, which induces fibrosis, provide a representative tumor microenvironment (62). Employing these advanced models is crucial for developing more accurate therapeutic strategies and predicting clinical outcomes in pancreatic cancer. Accordingly, we screened subcutaneous tumor models derived from cancer cell lines that exhibit the so-called stromal vessel type architecture, in which blood vessels are predominantly confined to the tumor stroma, and identified tumors formed by Capan-1 and BxPC-3 cells based on CD31/α-SMA staining (42).

Conventional CAR-T cell therapy showed potent antitumor effects in Panc-1 and AsPC-1-CD44v6 subcutaneous xenograft tumor models with scant stroma, but not in SU86.86 subcutaneous xenograft tumor models with abundant stroma (**Figures 4A–C**). Although CAR-T cells were present in the SU86.86 subcutaneous xenograft tumor, they were trapped within the abundant stromal tissue, preventing their effective delivery to cancer cells (**Figure 4E**). This suggests that stromal barriers limit the effectiveness of CAR-T cell therapy. Notably, RLN2-secreting CAR-T cell therapy demonstrated potent antitumor efficacy in SU86.86 xenograft tumors, overcoming resistance by breaching the stromal barrier and effectively targeting cancer cells. This underscores the potential applicability of CAR-T cell therapies addressing the physical stromal barriers in solid tumors.

In SU86.86 xenograft tumors, the enhanced antitumor effect of RLN2-secreting CAR-T cells appeared to be primarily due to the reduction in collagen type IV, likely mediated by RLN2-induced MMP-9 and MMP-7 expression (**Figure 5**). MMP-9 predominantly targets collagen type IV, laminin, and proteoglycans, the major components of the basement membrane, as well as denatured collagens. MMP-7 has a broader range of substrates than MMP-9 and targets various extracellular matrix components and membrane-bound proteins (64).

Following treatment with RLN2-secreting CAR-T cells, type IV collagen expression in the tumor was significantly reduced by RLN2-induced MMP-9 and MMP-7 expression, potentially facilitating easier contact between the extravasated CAR-T and cancer cells after traversing the interstitial stroma. Similarly, a previous study demonstrated that RLN2 primarily induces the degradation of collagen type IV in the basement membrane of tumors, promoting the infiltration of endogenous T cells (65). These findings indicate that RLN2-induced MMP9 and MMP7 expression may contribute to the observed reduction in collagen IV; however, further studies using receptor knockout or MMP inhibition models are required to establish a direct causal link.

Among the collagen types examined, the treatment only caused a clear reduction in collagen type IV—a key structural component of the basement membrane and major regulator of intratumoral interstitial fluid pressure. This may have contributed to decreased interstitial fluid pressure, thereby promoting tumor blood vessel permeability (66). These effects are hypothesized to have disrupted the physical stromal barrier and enhanced the antitumor activity of RLN2-secreting CAR-T cells in the SU86.86 subcutaneous xenograft tumor model.

In this study, we focused on the MMP-inducing effects of RLN2 and the associated collagen degradation using a subcutaneous xenograft tumor model in immunodeficient NSG mice. However, RLN2 may also exert additional effects beyond MMP induction during CAR-T cell therapy. For example, RLN2 can promote vasodilation by acting directly on endothelial cells. In previous reports using RLN2-overexpressing prostate cancer xenograft models, an increased number of vascular endothelial cells expressing factor VIII was observed in tumor tissues (67). While these findings suggest that RLN2 may influence the tumor vasculature, the functional significance of this effect in the context of cancer remains unclear.

The anti-apoptotic properties of RLN2 suggest that it may help CAR-T cells evade activation-induced cell death after binding to the target antigen *in vivo* (68, 69). Although these aspects require further investigation, RLN2 can potentially improve the efficacy of CAR-T cell therapy beyond the destruction of the physical stromal barrier.

Our study has some limitations. We demonstrated the utility of RLN2-secreting CAR-T cell therapy in an SU86.86-derived subcutaneous xenograft tumor model. Unlike the SU86.86 subcutaneous tumors, the Capan-1 and BxPC-3 subcutaneous tumor models—with histologies more closely resembling human pancreatic cancer—did not show improved antitumor efficacy with RLN2-secreting CAR-T cell therapy. These findings suggest that further enhancement of intratumoral stromal remodeling capacity in CAR-T cells may be useful for clinical translation to stroma-rich solid tumors.

Histological examination of preoperative pancreatic cancer resection specimens from our facility revealed that the staining pattern for collagen type IV in the basement membrane surrounding the neoplastic glands was similar to that observed in the SU86.86 subcutaneous xenograft tumor model. However, the interstitial matrix, consisting of collagen types I and III, was much thicker, generating a considerable distance between the tumor blood vessels and cancer clusters (data not shown). Therefore, hyaluronidase was used to evaluate tumor models that exhibited tissue characteristics similar to those of clinical samples. Hyaluronidase targets the abundant hyaluronic acid in the interstitial matrix (9, 70) or collagenase enzymes that degrade collagen types I and III to reduce tumor interstitial pressure (71). This approach allows CAR-T cells to reach cancer cell clusters more efficiently once extravasated from the tumor blood vessels. Additionally, the efficacy of CAR-T cells can be improved by enhancing RLN2 secretion.

In conclusion, RLN2 secreted by engineered CAR-T cells demonstrated specific MMP-inducing capabilities, promoted collagen degradation *in vivo*, and improved tumor suppression in xenograft models with abundant stroma. Our study indicated that incorporating RLN2 into CAR-T cells enhances their infiltration into solid tumors, providing a promising strategy that warrants further testing in other research models. We believe that our approach utilizing RLN2 to overcome the stromal barriers hindering CAR-T cell delivery to cancer cells represents a significant advancement in pancreatic and biliary cancer therapies, the efficacies of which have been limited.

## Data Availability

The original contributions presented in the study are included in the article/**Supplementary Material**. Further inquiries can be directed to the corresponding author.
