# Supplementary material for: Relaxin-2-secreting CAR-T cells exhibit enhanced efficacy in stromal-rich xenograft tumors
Source: Front Immunol. 2025 Jul 1;16:1506204. doi: 10.3389/fimmu.2025.1506204 (PMC12259638; doi:10.3389/fimmu.2025.1506204)
Supplement: Supplementary file 1 [file DataSheet1.pdf]

## Supplementary Figure Captions

**Supplementary Figure 1.** CAR-T cell manufacturing protocol and assessment of gene transduction efficiency.

**(A)** Schematic illustration of CD44v6 chimeric antigen receptor (CAR) plasmid construct, including the structure, gene insertion, and replacement strategy.

**(B)** Overview of CAR-T cell manufacturing protocol.

**(C)** Representative flow cytometric analysis of CAR gene transduction efficiency using a construct encoding enhanced green fluorescent protein (eGFP). eGFP-positive cells were stained with a PE-conjugated Fc gamma-specific antibody. Double-positive cells ($eGFP^+$/Fcγ $Ab^+$) were used to estimate CAR transduction efficiency.

**(D)** Transduction efficiency of $CD4^+$ and $CD8^+$ T cells before and after puromycin selection, quantified using flow cytometry of a PE-conjugated Fc gamma-specific antibody.

**(E)** Total CAR-T cell yield and CAR-positive cell frequency derived from peripheral blood of three independent donors. CAR expression was determined using Fc gamma-specific antibody staining and flow cytometry.

**Supplementary Figure 2.** CD44v6 expression in various human cancer cell lines.

**(A)** Representative flow cytometric analysis of CD44v6 surface expression in multiple cancer cell lines. Cells were stained with an anti-CD44v6 monoclonal antibody (clone VFF-18; brown) or isotype control antibody (mouse IgG1; blue). A rightward shift in fluorescence intensity in CD44v6-positive cell lines indicates specific binding of the CD44v6 antibody.

**(B)** Western blot analysis of CD44v6 expression using the same antibody clone (VFF-18) across the indicated cancer cell lines. Multiple bands (arrowheads) were detected in CD44v6-positive cell lines, which may reflect post-translational modifications such as glycosylation.

**Supplementary Figure 3.** Phenotypic and functional comparison between conventional and RLN2-secreting CAR-T cells derived from donor 1 peripheral blood mononuclear cells (PBMCs).

**(A)** In vitro cytotoxicity of conventional and RLN2-secreting CAR-T cells against SU86.86, Panc-1, and AsPC-1-CD44v6 cells. CAR-T cells (effectors) were co-cultured with cancer cells (targets) at multiple effector-to-target (E:T) cell ratios (0.25:1–4:1) for 3 d to assess dose-dependent cytolytic activity. Cancer cell survival was measured using crystal violet assays. Absorbance at 590 nm ($A_{590}$) was recorded and normalized to untreated control wells to calculate relative cell viability. Data are presented as mean ± standard deviation (SD) from three independent experiments performed in triplicate. ns, not significant [two-way analysis of variance (ANOVA)].

**(B)** Memory and effector subset distribution in conventional and RLN2-secreting CAR-T cells following co-culture. CAR-T cells were incubated with or without the indicated target cell lines at an E:T ratio of 2:1 (i.e., two CAR-T cells per target cancer cell) in cytokine-depleted medium for 3 d. After co-culture, cells were stained with CD62L and CD45RO and gated on $CD45^{+}CD3^{+}$ CAR-T cells for flow cytometric analysis.

**(C)** Cytokine and RLN2 secretion profiles of conventional and RLN2-secreting CAR-T cells. Supernatants were collected after 48 h of co-culture at an E:T ratio of 4:1, with or without the indicated cell lines. Tumor necrosis factor α (TNF-α), interferon γ (IFN-γ), and RLN2 levels were measured using enzyme-linked immunosorbent assay (ELISA). Data are presented as mean ± SD from three independent experiments conducted in triplicate. $^{**}P < 0.01$; ns, not significant (unpaired *t*-tests).

**Supplementary Figure 4.** Phenotypic and functional comparison between conventional and RLN2-secreting CAR-T cells derived from the PBMCs of donors 2 and 3.

**(A)** In vitro cytotoxicity of conventional and RLN2-secreting CAR-T cells against SU86.86, Panc-1, and AsPC-1-CD44v6 cells. CAR-T cells (effectors) derived from PBMCs were co-cultured with the indicated cancer cell lines (targets) at multiple E:T ratios (0.25:1–4:1) for 3 d to evaluate dose-dependent cytolytic activity. Cancer cell survival was assessed using a crystal violet assay. Absorbance at 590 nm ($A_{590}$) was measured and normalized to untreated control wells to calculate relative viability. Data are presented as mean ± SD from three independent experiments performed in triplicate. ns, not significant (two-way ANOVA).

**(B)** Memory and effector subset distribution in conventional and RLN2-secreting CAR-T cells following co-culture. CAR-T cells derived from donors 2 and 3 were co-cultured with or without the indicated target cancer cell lines at an E:T ratio of 2:1 in cytokine-depleted medium for 3 d. After co-culture, cells were stained with CD62L and CD45RO

and gated on $CD45^+CD3^+$ CAR-T cells for flow cytometric analysis. Representative scatter plots are shown for each donor and target cell condition.

**Supplementary Figure 5.** Antitumor effects of CD44v6 CAR-T cell therapy in AsPC-1 subcutaneous xenograft tumor model.

**(A)** Histological analysis of AsPC-1 xenograft tumors. Formalin-fixed paraffin-embedded tumor sections were stained for CD44v6 expression. Scale bar = 200 µm.

**(B)** In vitro cytotoxicity of donor 1-derived CD44v6 CAR-T cells against AsPC-1 cells. CAR-T cells were co-cultured with AsPC-1 cells for 3 d, and cancer cell viability was quantified using a crystal violet assay. Absorbance at 590 nm ($A_{590}$) was measured and normalized to untreated control wells. Data are presented as mean ± SD from three independent experiments performed in triplicate. ns, not significant (two-way ANOVA).

**(C)** In vivo antitumor effect of CD44v6 CAR-T cell therapy against AsPC-1 xenograft tumors. Mice received a single intravenous injection of either control T (Ctrl-T; $1 \times 10^7$ cells, $n = 6$) or CD44v6 CAR-T cells ($1 \times 10^7$ cells, $n = 6$). Data are presented as mean ± SD. ns, not significant (two-way ANOVA).

**(D)** In vivo tracking of Luc2-expressing CAR-T cells in AsPC-1 xenograft tumors. A total of $1 \times 10^7$ Luc2-expressing CAR-T cells were injected into the tail vein, and bioluminescence was measured using the IVIS imaging system on days 7 and 14 post-injection.

**Supplementary Figure 6.** MMP expression in xenograft tumors treated with CAR-T or RLN2-secreting CAR-T cells. The same samples shown in Figure 4 were used to measure the expression of MMP-1, -2, -3, -13, and -14. CAR T cell group ($n = 9$, day 3; $n = 5$, day 7), RLN2-secreting CAR T cell group ($n = 7$, day 3; $n = 7$, day 7). The results from two independent experiments are shown (mean ± SD). ns, not significant (compared between CAR-T and RLN2-secreting CAR-T cells, day 3 and day 7, after treatment, using an unpaired *t*-test).

**Supplementary Figure 7.** Antitumor effects of RLN2-secreting CD44v6 CAR-T cell therapy in Capan-1 and BxPC-3 subcutaneous xenograft tumor models.

**(A)** Representative flow cytometry histograms showing CD44v6 expression in Capan-1 and BxPC-3 cells. Cells were stained with anti-CD44v6 antibody (clone VFF-18;

brown) or isotype control antibody (mouse IgG1, blue). A rightward shift in fluorescence intensity indicates positive CD44v6 expression in both cell lines.

**(B)** Western blot analysis of the RLN2 receptor LGR7/RXFP1 in Capan-1 and BxPC-3 cells. GAPDH was used as loading control.

**(C)** Immunofluorescence staining of vascular and stromal markers in Capan-1 and BxPC-3 xenograft tumors. Endothelial cells were labeled with anti-CD31 (green), stromal cells (including myofibroblasts and pericytes) with anti-α-smooth muscle actin (α-SMA, red), and nuclei with DAPI (blue). Scale bar = 100 µm.

**(D)** In vitro cytotoxicity (left) and in vivo antitumor activity (right) of donor 3-derived conventional CD44v6 CAR-T cells against Capan-1 and BxPC-3 tumor models.

Left: CAR-T cells (effectors) were co-cultured with target cancer cells at various E:T ratios (0.25:1–4:1) for 3 d. Cytotoxicity was assessed using a crystal violet assay, and absorbance at 590 nm ($A_{590}$) was measured and normalized to untreated control wells. Data are presented as the mean ± SD from three independent experiments performed in triplicate. ***$P < 0.001$ (two-way ANOVA).

Right: Tumor-bearing mice received a single intravenous injection of either Ctrl-T ($1 \times 10^7$ cells, $n = 6$) or CD44v6 CAR-T cells ($1 \times 10^7$ cells, $n = 6$). Tumor volume was monitored over time. Data are presented as mean ± SD. ns, not significant (two-way ANOVA).

**(E)** In vitro cytotoxicity (left) and in vivo antitumor activity (right) of donor 3-derived conventional and RLN2-secreting CD44v6 CAR-T cells against Capan-1 and BxPC-3 xenograft tumor models.

Left: CAR-T cells were co-cultured with target cells for 3 d, and cytolytic activity was assessed using a crystal violet assay. Absorbance at 590 nm ($A_{590}$) was normalized to untreated controls. Data are presented as mean ± SD from three independent experiments performed in triplicate. ns, not significant (two-way ANOVA).

Right: Tumor-bearing mice were intravenously injected once with either Ctrl-T ($1 \times 10^7$ cells, $n = 6$), conventional CD44v6 CAR-T ($n = 6$), or RLN2-secreting CD44v6 CAR-T cells ($n = 6$). Tumor growth was monitored over time. Data are presented as mean ± SD. **$P < 0.01$; ns, not significant (two-way ANOVA).
