# Supplementary material for: Relaxin-2-secreting CAR-T cells exhibit enhanced efficacy in stromal-rich xenograft tumors
Source: Front Immunol. 2025 Jul 1;16:1506204. doi: 10.3389/fimmu.2025.1506204 (PMC12259638; doi:10.3389/fimmu.2025.1506204)
Supplement: Supplementary file 2 [file DataSheet2.pdf]

Table

Table 1. PCR primer sequences.

MMP-1 (F)	AAAATTACACGCCAGATTTGCC
MMP-1 (R)	GGTGTGACATTACTCCAGAGTTG
MMP-2 (F)	CCCACTGCGGTTTTCTCGAAT
MMP-2 (R)	CAAAGGGGTATCCATCGCCAT
MMP-3 (F)	CACTCACAGACCTGACTCGGTT
MMP-3 (R)	AAGCAGGATCACAGTTGGCTGG
MMP-7 (F)	GGTCACCTACAGGATCGTATCATAT
MMP-7 (R)	CATCACTGCATTAGGATCAGAGGAA
MMP-9 (F)	TTCCAAACCTTTGAGGGCGA
MMP-9 (R)	CAAAGGCGTCGTCAATCACC
MMP-13 (F)	CCTTGATGCCATTACCAGTCTCC
MMP-13 (R)	AAACAGCTCCGCATCAACCTGC
MMP-14 (F)	CGCTACGCCATCCAGGGTCTCAAA
MMP-14 (R)	CGGTCATCATCGGGCAGCACAAAA
human GAPDH (F)	AACGGGAAGCTTGTCATCAA
human GAPDH (R)	TGGACTCCACGACGTACTCA
