# Supplementary figures and images for: Relaxin-2-secreting CAR-T cells exhibit enhanced efficacy in stromal-rich xenograft tumors
Source: Front Immunol. 2025 Jul 1;16:1506204. doi: 10.3389/fimmu.2025.1506204 (PMC12259638; doi:10.3389/fimmu.2025.1506204)

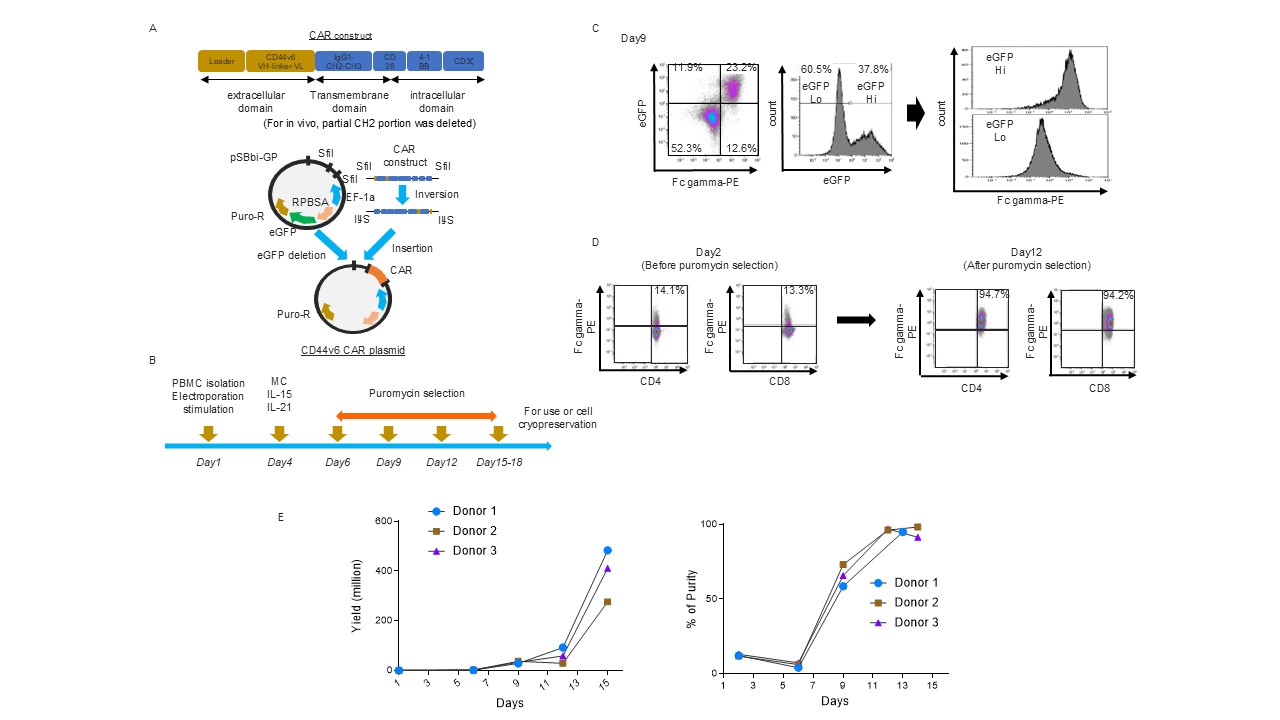

Supplement: Supplementary file 3 [file Image1.jpeg]

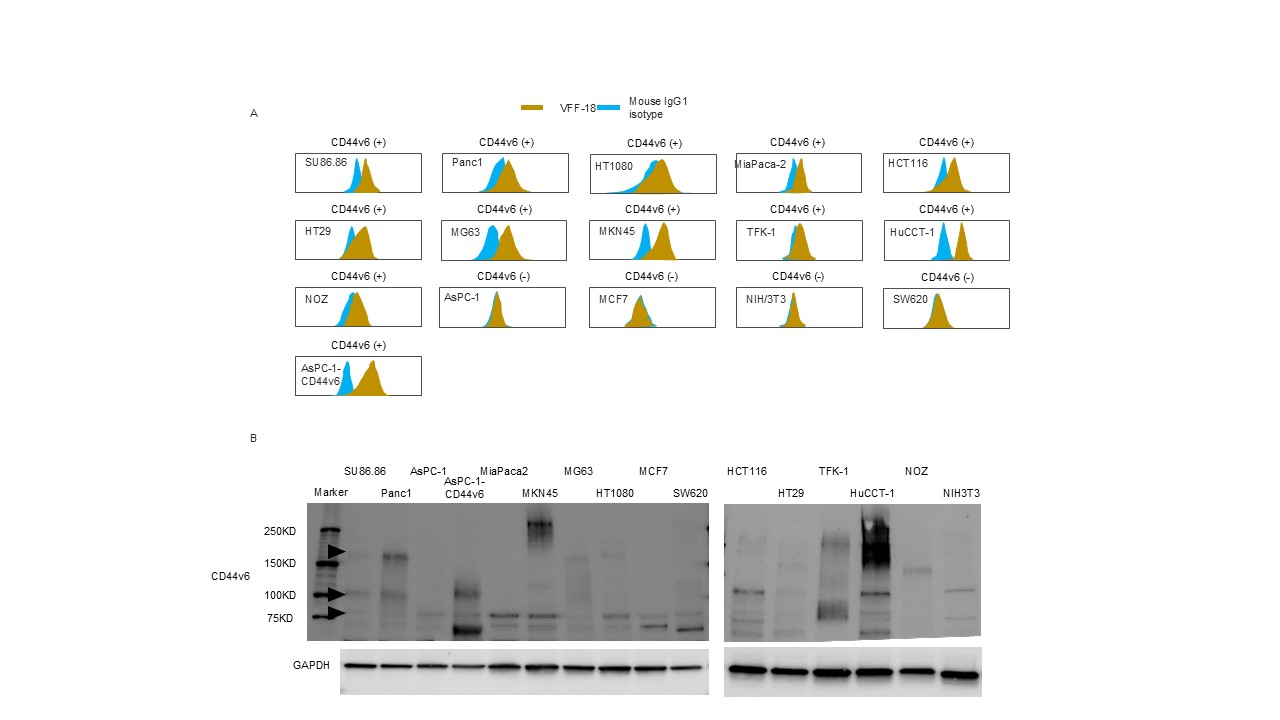

Supplement: Supplementary file 4 [file Image2.jpeg]

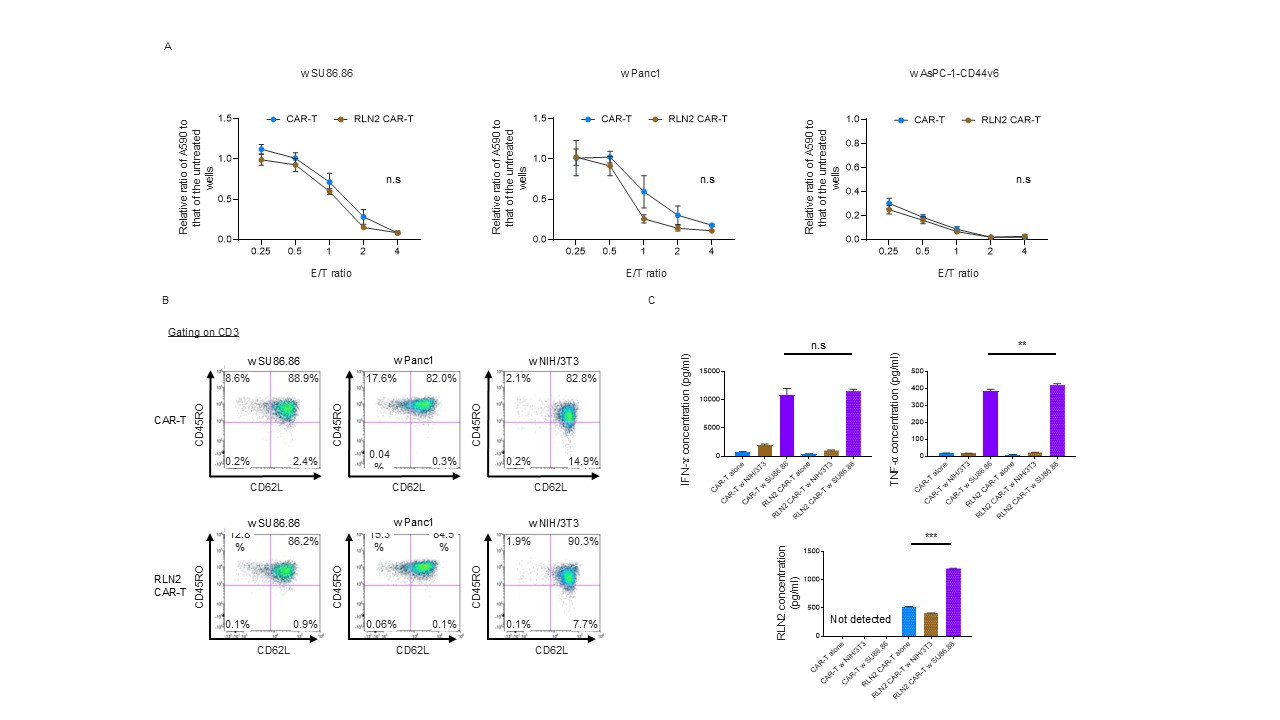

Supplement: Supplementary file 5 [file Image3.jpeg]

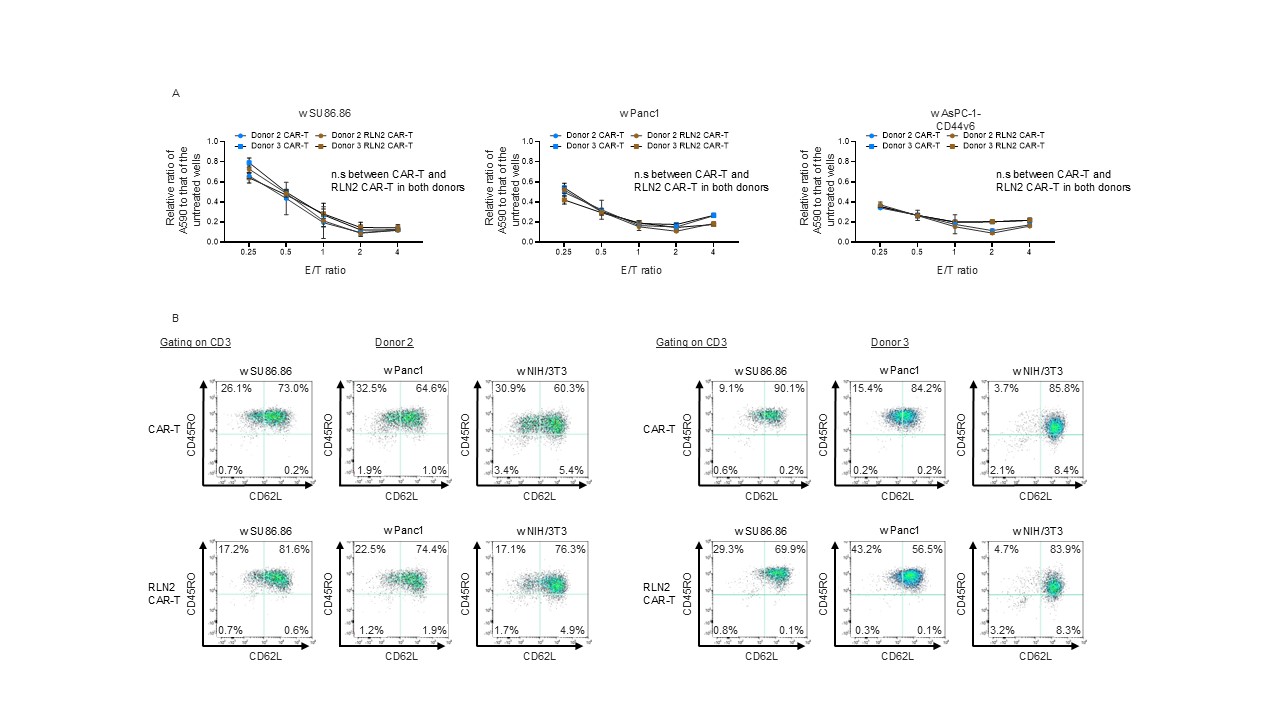

Supplement: Supplementary file 6 [file Image4.jpeg]

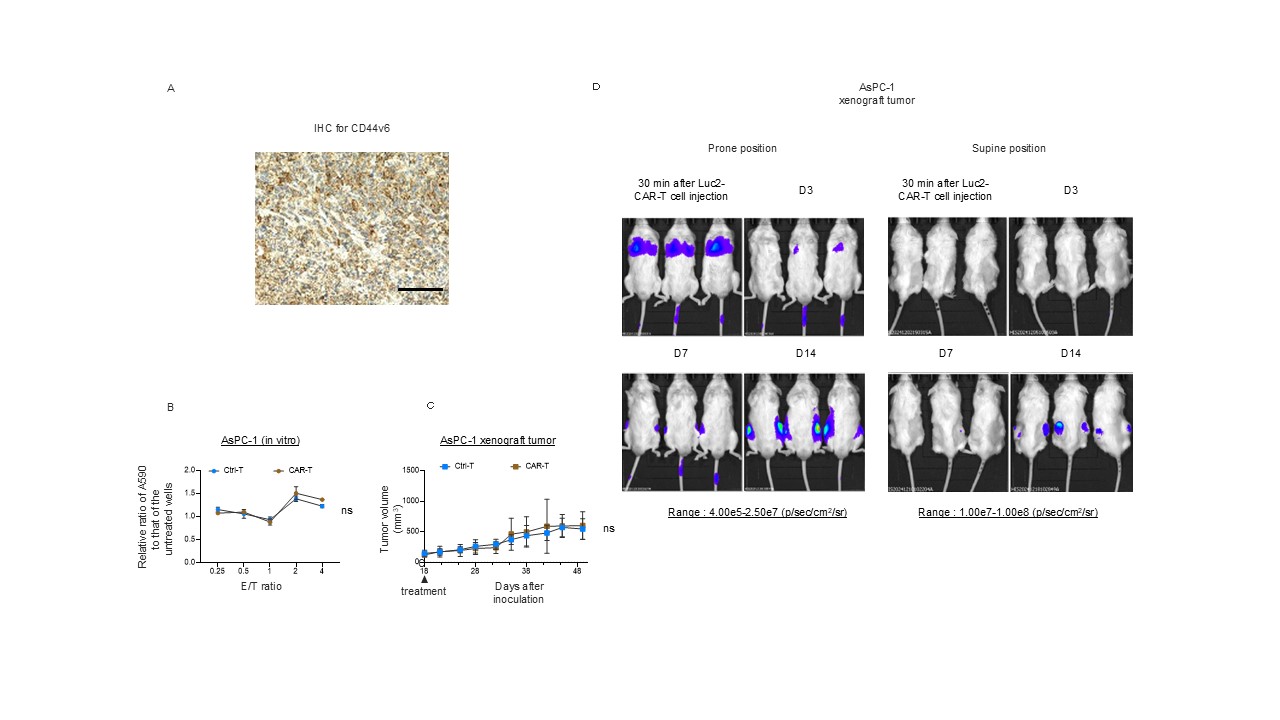

Supplement: Supplementary file 7 [file Image5.jpeg]

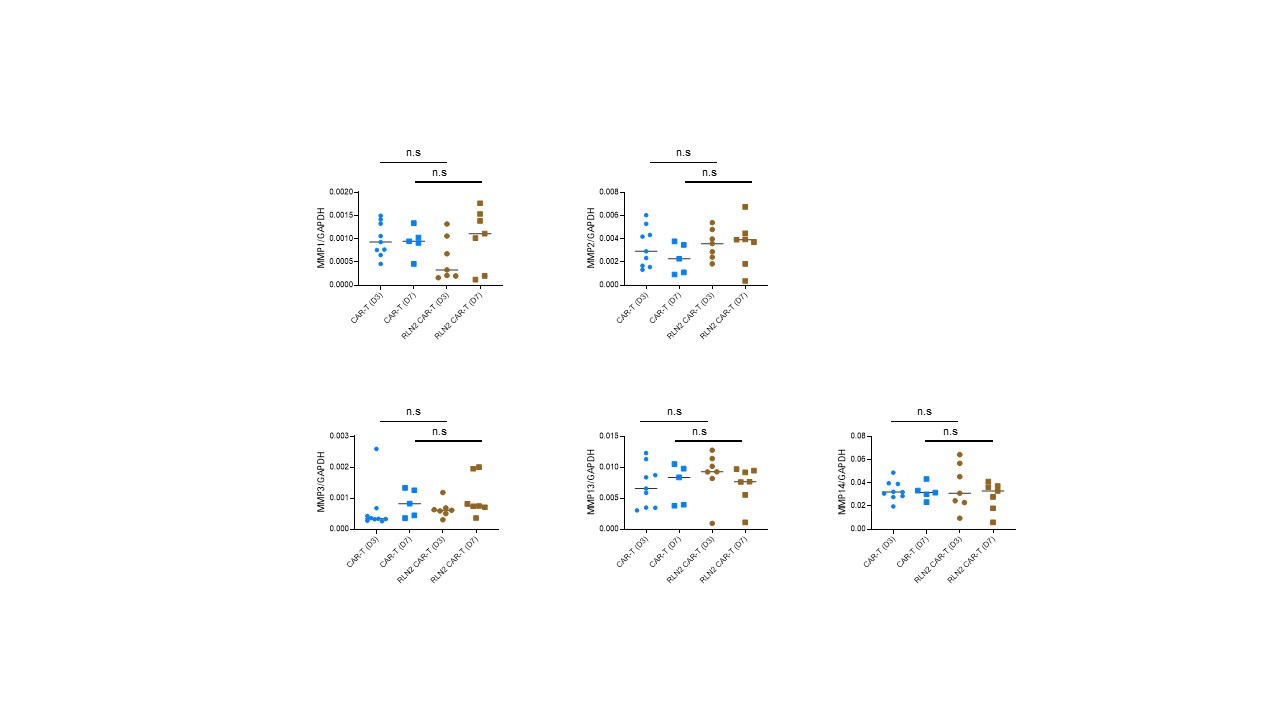

Supplement: Supplementary file 8 [file Image6.jpeg]

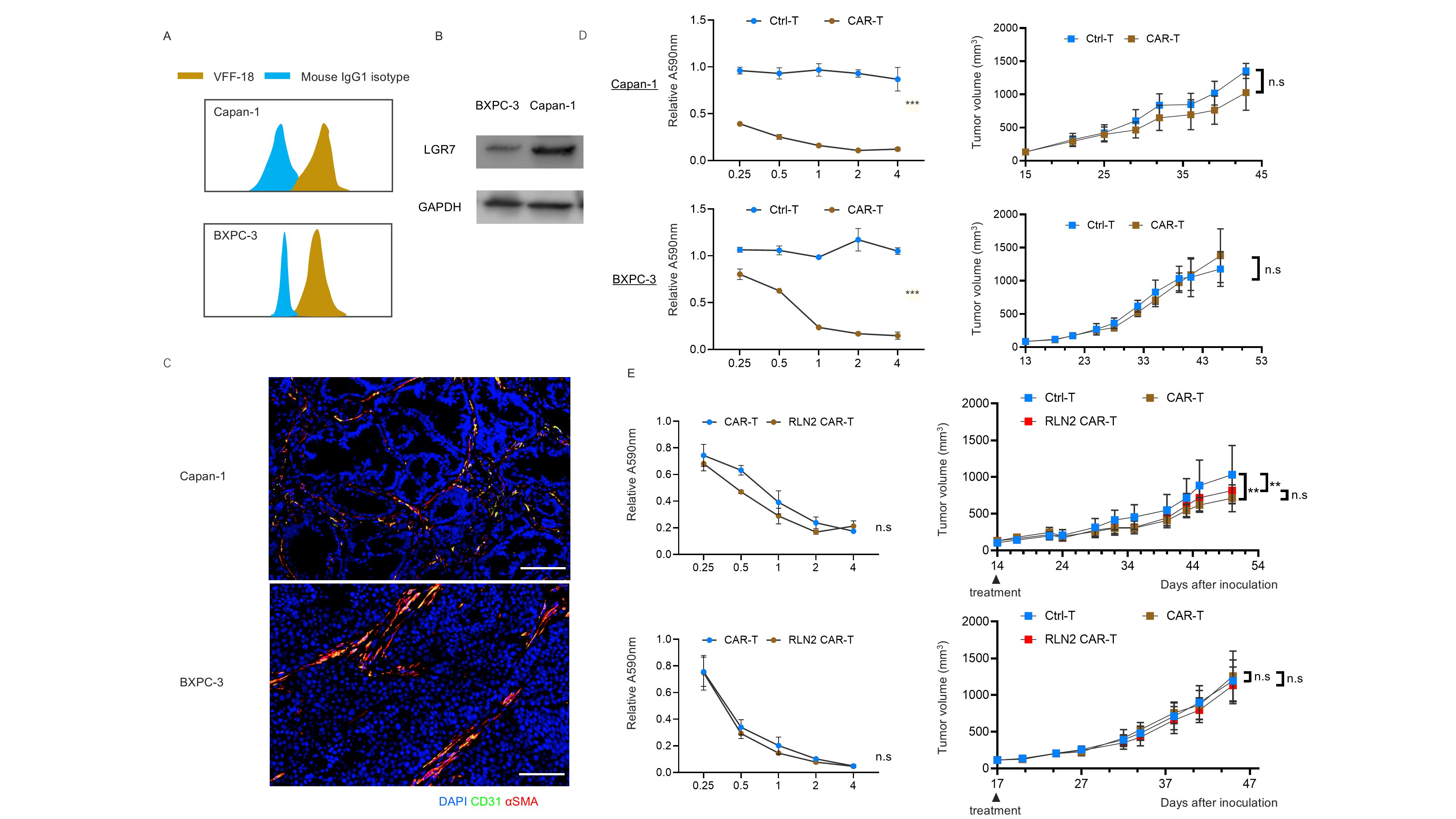

Supplement: Supplementary file 9 [file Image7.jpeg]
